# The Challenges of Caring for People Dying From COVID-19: A Multinational, Observational Study (CovPall)

**DOI:** 10.1016/j.jpainsymman.2021.01.138

**Published:** 2021-09

**Authors:** Adejoke O. Oluyase, Mevhibe Hocaoglu, Rachel L. Cripps, Matthew Maddocks, Catherine Walshe, Lorna K. Fraser, Nancy Preston, Lesley Dunleavy, Andy Bradshaw, Fliss E.M. Murtagh, Sabrina Bajwah, Katherine E. Sleeman, Irene J. Higginson, CovPall study team

**Affiliations:** 1 Cicely Saunders Institute of Palliative Care, Policy and Rehabilitation, King's College London, London, UK; 2 International Observatory on End of Life Care, Division of Health Research, Lancaster University, Lancaster, UK; 3 Health Sciences, University of York, York, North Yorkshire, UK; 4 Wolfson Palliative Care Research Centre, Hull York Medical School, University of Hull, Hull, UK; 5 King's College Hospital NHS Foundation Trust, Denmark Hill, UK

**Keywords:** Palliative care, COVID-19, pandemics, severe acute respiratory syndrome coronavirus 2, end of life care, hospices

## Abstract

**Context:**

Systematic data on the care of people dying with COVID-19 are scarce.

**Objectives:**

To understand the response of and challenges faced by palliative care services during the COVID-19 pandemic, and identify associated factors.

**Methods:**

We surveyed palliative care and hospice services, contacted via relevant organizations. Multivariable logistic regression identified associations with challenges. Content analysis explored free text responses.

**Results:**

A total of 458 services responded; 277 UK, 85 rest of Europe, 95 rest of the world; 81% cared for patients with suspected or confirmed COVID-19, 77% had staff with suspected or confirmed COVID-19; 48% reported shortages of Personal Protective Equipment (PPE), 40% staff shortages, 24% medicines shortages, 14% shortages of other equipment. Services provided direct care and education in symptom management and communication; 91% changed how they worked. Care often shifted to increased community and hospital care, with fewer admissions to inpatient palliative care units. Factors associated with increased odds of PPE shortages were: charity rather than public management (OR 3.07, 95% CI 1.81–5.20), inpatient palliative care unit rather than other settings (OR 2.34, 95% CI 1.46–3.75). Being outside the UK was associated with lower odds of staff shortages (OR 0.44, 95% CI 0.26–0.76). Staff described increased workload, concerns for their colleagues who were ill, whilst expending time struggling to get essential equipment and medicines, perceiving they were not a front-line service.

**Conclusion:**

Palliative care services were often overwhelmed, yet felt ignored in the COVID-19 response. Palliative care needs better integration with health care systems when planning and responding to future epidemics/pandemics.

**Key message**

Palliative care teams actively supported symptomatic and dying patients with COVID-19, and their families. However, they felt ignored by national and international pandemic responses and often lacked equipment, staff, medicines, integration and recognition.

Alt-text: Unlabelled box

## Introduction

COVID-19 evolved from a mystery illness to a pandemic in 93 days, overwhelming services in many countries.^1^ The World Health Organization rapidly issued guidance on maintaining essential health services during the pandemic, highlighting prevention, maternity, emergency care and chronic diseases, without mention of palliative care.^2^ COVID-19 has an overall case fatality ratio between 1% and 4%;^3^ by January 2021 there were over 2.1 million confirmed COVID-19 deaths worldwide.^4^

Palliative care is multidisciplinary, holistic and person-centered treatment, care and support for people with life-limiting illness, and those important to them, such as family and friends. It is recommended in respiratory^5^^,^^6^ and infectious diseases,^7^ and in recent guidance from the European Respiratory Society in COVID-19.^8^^,^^9^ In the COVID-19 pandemic, palliative care has an important role in ensuring symptom control, training of nonspecialists in symptom management and care of dying patients, compassionate communication, psychosocial support for patients, carers and health care professionals, advance care planning and bereavement support,^8^^,^^10^ supporting patients wherever they want to be cared for (Table 1).^11^

**Table 1:** Palliative Care Services Provided in Different Settings

Palliative Care Setting^a^	Description of Setting
Inpatient palliative care unit	Provides specialist inpatient palliative care. It can be a ward within, or adjacent to, a hospital, or a free standing building. In some countries, it is called an inpatient hospice.
Hospital palliative care team	Provides specialist palliative care advice and support to other clinical staff, patients and their families and carers in the hospital environment. They offer formal and informal education and liaise with other services in and out of the hospital.
Home palliative care team	Provides specialist palliative care to patients who need it at home, or in care homes or residential homes, and support their families and carers. They also provide specialist advice to general practitioners, family doctors and nurses caring for the patients.
Home nursing	Provides intensive home nursing care for the patient at home, sometimes referred to as hospice or hospital at home, often supporting patients whose care needs are such that without this they would be admitted to an inpatient palliative care unit or hospital.

a Palliative care and hospice services have multiprofessional teams of dedicated staff trained in palliative care, comprising doctors, nurses, and often social workers and therapists. They provide expertise in pain and symptom management, holistic and psychosocial care, decision making, advance care planning, end of life care and often bereavement support. They include support in the settings described above (one service may provide support in one or more settings).

The only review published on the role and response of palliative care and hospice services during pandemics included 10 observational studies^12^ from single units or countries: West Africa, Taiwan, Hong Kong, Singapore, the U.S. (a simulation), and Italy (the only one considering COVID-19). The review concluded hospice and palliative care services are essential in the response to COVID-19 but systematic data are urgently needed to inform how to improve care for those who are likely to die, and/or have severe symptoms.

Palliative care services are often managed separately from other medical services and may be exceptionally vulnerable to disruption in pandemics as they often rely on charity funding. During the COVID-19 pandemic there were media reports of acute shortages of Personal Protective Equipment (PPE) and medicines that limited care.^13^ There is little systematic data about these situations.^12^^,^^14^ This study aimed to understand the response of and challenges faced by palliative care services during the COVID-19 pandemic, and to identify factors associated with challenges experienced, in particular shortages of equipment, medicines and staff. We tested two a priori null hypotheses:

- There are no differences in shortages between services with different management types (e.g., charity and public)
- There are no differences in shortages between settings; e.g., between hospital based and nonhospital based; or community and noncommunity settings.

## Methods

### Study Design and Participants

CovPall is a multicenter multinational observational study of palliative care during the COVID-19 pandemic. This paper reports an on-line survey of palliative care services, the first main component of CovPall. The survey opened on April 23rd, 2020 and closed July 31st, 2020. The survey received ethical (Institutional Review Board) approval from King's College London Research Ethics committee (LRS-19/20-18541); study sponsor: King's College London, co-sponsor: King's College Hospital NHS Foundation Trust, registered ISRCTN 16561225. It is reported according to STROBE,^15^ CHERRIES^16^ and MORECARE^17^statements.

Inclusion criteria: Any palliative care service (Table 1)^11^; caring for adults and/or children; managed by charity, public, private or other sector. Exclusion criteria: not a specialist palliative care/hospice service,e.g., no staff with specific expertise/training in palliative care.

### Procedures and Questionnaire

Services were identified and contacted through national and multinational palliative care and hospice organizations (supplementary file, box S1) and provided with a link to the on-line survey and the participant information sheet. Completion indicated consent. The questionnaire was developed and piloted, building on an earlier survey of Italian hospices,^14^ adding questions on the impact of and response to COVID-19. It was intended to be brief, taking ∼30 minutes to complete. Free-text comments were invited using extension (e.g., other, please specify), expansion (e.g., elaborating on closed responses by giving details or reasons), and general (e.g., any other comments) approaches^18^ (see Appendix II). Free text responses were planned to shed light on the closed text responses, provide a richer context with specific examples, and as a “safety net” to identify issues which might have been missed by the closed questions. Data were anonymized before analysis.

### Sample Size and Analysis

We aimed to have responses from >390 services, ∼130 inpatient palliative care units, 130 hospital palliative care teams, and 130 home palliative care teams. Subgroups of this size (>105) are sufficient to detect differences with effect sizes of 0.35, using χ2 (*P <* .05, df = 5, power 80%).

After removing duplicate and ineligible entries all available data were analyzed. We report completion rate and summary statistics. Missing data were not imputed. We used contingency tables, χ2 tests, correlations and multivariable logistic regression to explore relationships between variables (using SPSS v26, STATA v16). We preselected four dependent variables critical to delivering care, presence or not of shortages of: PPE, staff, medicines, and other equipment (such as syringe drivers). Independent variables were: country/region, charity or public management, settings (in-patient palliative care unit, hospital palliative care team, home palliative care team, home nursing), experiences with COVID-19 and level of busyness; criteria for inclusion in multiple regression analyses was *P <* .10 in univariable analysis. We excluded variables exhibiting collinearity with independent variables already included if the variance inflation factor >10 or chi-square test, *P <* .05.

Free text comments were explored in Excel using content analysis to understand the impact of COVID-19 and the strategies, enablers and actions deployed by services.

## Results

In total, 489 questionnaires were commenced, 477 completed (completion rate 97.5%); of these 15 were duplicates and 2 triplicates of entries with the same name/email; 2 were invalid being from one researcher without a palliative care service, leaving 458 valid responses: 277 UK, 85 rest of Europe, 95 rest of the world, 1 missing country (Table 2; supplementary Table S1). Services were usually publicly (204, 46.4%), or charity managed (192, 43.6%); 19 (4.3%) were privately managed, 25 (5.7%) other; 18 missing. Charity managed services reported less integration with national health services (mean [SD]: 67.82 [19.75]) compared to publicly managed services (mean [SD]: 75.10 [19.75]), (mean difference [95% CI]: 7.28 [3.24–11.33]; *P <* .001). Overall, 261 services provided inpatient palliative care units, 261 home care teams, 217 hospital palliative care teams, and 119 home nursing teams. Many services offered care in more than one setting (supplementary Table S2).

**Table 2:** Characteristics of Responding Palliative Care and Hospice Services by Region

			Rest of the world (*n* = 95)			
	UK (*n* = 277)	Rest of Europe (*n* = 85)	LIC/LMIC (*n* = 17)	UMIC (*n* = 19)	HIC (*n* = 59)	Total
**Management type (*n*/*N*, %)**^a^						
Charitable / nonprofit	143/262 (54.6%)	23/85 (27.1%)	5/17 (29.4%)	6/17 (35.3%)	14/58 (24.1%)	192/440 (43.6%)^b^
Public	103/262 (39.3%)	51/85 (60%)	8/17 (47.1%)	6/17 (35.3%)	36/58 (62.1%)	204/440 (46.4%)^b^
Private	1/262 (0.4%)	9/85 (10.6%)	2/17 (11.8%)	5/17 (29.4%)	2/58(3.4%)	19/440 (4.3%)^b^
Other	15/262 (5.7%)	2/85 (2.4%)	2/17 (11.8%)	-	6/58 (10.3%)	25/440 (5.7%)^b^
Missing	15	-	-	2	1	18
**Setting (*n*/*N*, %)**						
Inpatient PC unit	168/277 (60.6%)	44/85 (51.8%)	8/17 (47.1%)	6/19 (31.6%)	34/59 (57.6%)	261/458 (57%)^b^
Hospital PC team	135/277 (48.7%)	26/85 (30.6%)	9/17 (52.9%)	10/19 (52.6%)	37/59 (62.7%)	217/458 (47.4%)^b^
Home PC team	160/277 (57.8%)	47/85 (55.3%)	10/17 (58.8%)	9/19 (47.4%)	34/59 (57.6%)	261/458 (57%)^b^
Home nursing	92/277 (33.2%)	15/85 (17.6%)	1/17 (5.9%)	4/19 (21.1%)	7/59 (11.9%)	119/458 (26%)^b^
Total	277	85	17	19	59	458^b^
**Experience with suspected or confirmed COVID-19**						
**Services with confirmed or suspected COVID-19 cases** (*n*/*N*, %)^a^	248/264 (93.9%)	60/83 (72.3%)	9/17 (52.9%)	7/17 (41.2%)	33/58 (56.9%)	358/440 (81.4%)^b^
Missing	13	2	-	2	1	18
**Approximate number of confirmed or suspected COVID-19 cases per service**						
Median (Q1, Q3)	25.5 (7, 70)	15 (4.5, 35.5)	3 (2, 70)	8 (2, 20)	6 (2, 11)	16 (5.5, 56)
Total	234	61	9	7	33	345
**Services with confirmed or suspected COVID-19 cases** (*n*/*N*, %)						
In-patient PC unit	146/158 (92.4%)	30/43 (69.8%)	3/8 (37.5%)	3/5 (60%)	20/34 (58.8%)	203/249 (81.5%)^b^
Hospital PC team	127/129 (98.4%)	20/26 (76.9%)	5/9 (55.6%)	4/9 (44.4%)	26/36 (72.2%)	182/209 (87.1%)
Home PC team	143/151 (94.7%)	35/46 (76.1%)	5/10 (50%)	3/7 (42.9%)	17/34 (50%)	204/249 (81.9%)^b^
Home nursing	82/87 (94.3%)	7/13 (53.8%)	0/1 (0%)	0/3 (0%)	5/7 (71.4%)	94/111 (84.7%)
**Severity of disease in patients with suspected or confirmed COVID-19** (*n*/*N*, %)						
Severely ill or dying due mainly to COVID-19	112/248 (45.2%)	13/60 (21.7%)	1/9 (11.1%)	0/7	5/33 (15.2%)	131/358 (36.6%)^b^
Pre-existing illnesses/comorbidities as well as COVID-19 who are severely ill or dying	192/248 (77.4%)	34/60 (56.7%)	3/9 (33.3%)	5/7 (71.4%)	15/33 (45.5%)	250/358 (69.8%)^b^
Patients known to service already who now have COVID-19	129/248 (52%)	21/60 (35%)	3/9 (33.3%)	2/7 (28.6%)	12/33 (36.4%)	168/358 (46.9%)^b^
**Services with staff with suspected or confirmed COVID-19** (*n*/*N*, %)^a^	238/262 (90.8%)	55/83 (66.3%)	4/16 (25%)	7/17 (41.2%)	30/58 (51.7%)	335/437 (76.7%)^b^
Missing	15	2	1	2	1	21
**Type of staff with suspected or confirmed COVID-19** (*n*/*N*, %)						
Nurses	224/238 (94.1%)	51/55 (92.7%)	3/4 (75%)	7/7 (100%)	22/30 (73.3%)	308/335 (91.9%)^b^
Physicians	161/238 (67.6%)	32/55 (58.2%)	2/4 (50%)	3/7 (42.9%)	15/30 (50%)	214/335 (63.9%)^b^
Allied health professionals, managed	92/238 (38.7%)	11/55 (20%)	3/4 (75%)	1/7 (14.3%)	7/30 (23.3%)	115/335 (34.3%)^b^
Reception/administrative staff	74/238 (31.1%)	9/55 (16.4%)	0/4	2/7 (28.6%)	3/30 (10%)	88/335 (26.3%)^b^
Managers	69/238 (29%)	8/55 (14.5%)	0/4	1/7 (14.3%)	0/30	78/335 (23.3%)^b^
Others	48/238 (20.2%)	5/55 (9.1%)	1/4 (25%)	0/7	4/30 (13.3%)	58/335 (17.3%)^b^
**Other support (*n*, %)**						
**Services that have encountered patients or families with COVID-19 who are from black and minority ethnic groups** (*n*/*N*, %)^a^	93/254 (36.6%)	16/76 (21.1%)	3/16 (18.8%)	5/16 (31.3%)	15/56 (26.8%)	132/419 (31.5%)^b^
Missing	23	9	1	3	3	39
**Shortages (*n*, %)**						
**Personal Protective Equipment (PPE) shortages** (*n*/*N*, %)^a^	129/258 (50%)	38/75 (50.7%)	9/15 (60%)	6/17 (35.3%)	19/54 (35.2%)	201/419 (48%)
Missing	19	10	2	2	5	39^b^
**PPE shortages in the last 7 days** (*n*/*N*, %)^c^	50/127 (39.4%)	7/38 (18.4%)	7/9 (77.8%)	5/6 (83.3%)	6/19 (31.6%)	75/199 (37.7%)
Missing or not applicable^d^	150	47	8	13	40	259^b^
**Key medicines shortages** (*n*/*N*, %)^a^	63/255 (24.7%)	19/73 (26%)	4/15 (26.7%)	4/17 (23.5%)	11/54 (20.4%)	101/414 (24.4%)
Missing	22	12	2	2	5	44^b^
**Key medicines shortages in the last 7 days** (*n*/*N*, %)^c^	28/63 (44.4%)	3/18 (16.7%)	4/4(100%)	4/4 (100%)	6/11 (54.5%)	45/100 (45%)
Missing or not applicable^d^	214	67	13	15	48	358^b^
**Equipment shortages (e.g. syringe drivers)** (*n*/*N*, %)^a^	45/256 (17.6%)	3/73 (4.1%)	4/14 (28.6%)	2/17 (11.8%)	2/54 (3.7%)	56/414 (13.5%)
Missing	21	12	3	2	5	44^b^
**Equipment shortages in the last 7 days** (*N*/*n*, %)^c^	19/42 (45.2%)	1/3 (33.3%)	3/3 (100%)	2/2 (100%)	2/2 (100%)	27/52 (51.9%)
Missing or not applicable^d^	235	82	14	17	57	406^b^
**Staff shortages** (*n*, %)^a^	117/255 (45.9%)	26/75 (34.7%)	4/14 (28.6%)	6/16 (37.5%)	13/54 (24.1%)	166/414 (40.1%)
Missing	22	10	3	3	5	44^b^
**Staff shortages in the last 7 days** (*N*/*n*, %)^c^	61/114 (53.5%)	9/26 (34.6%)	3/4 (75%)	5/5 (100%)	5/13 (38.5%)	83/162 (51.2%)
Missing or not applicable^d^	163	59	13	14	46	296^b^

Note: UK = United Kingdom, Rest of Europe excludes UK, LIC = low-income countries, LMIC = lower middle-income countries, UMIC = upper middle-income countries, HIC = high-income countries, NHS = National Health Service, PC = palliative care.

a N of value and valid *N* denominator are provided. Percentages are of valid values, unless otherwise stated. Number of missing responses for each category is provided.

b Includes data from the one missing country.

c Response for “yes” and “sometimes” both coded as “yes”.

d Not applicable includes those services who did not have shortages.

### Overall Impact of COVID-19 on Palliative and Hospice Services

Of all responding services, 91% changed how they worked as a result of COVID-19; 77% had staff who had suspected or confirmed cases of COVID-19. 81% of services had cared for patients with suspected or confirmed COVID-19, or both; of these, three main groups were cared for: patients with pre-existing illness or morbidities who were severely ill or dying from COVID-19 not previously known to palliative care (70% of services); patients dying from COVID-19 already known to palliative care services (47%); and patients severely ill or dying from COVID-19 but without pre-existing illness or morbidities (37%) (Table 2, supplementary Table S1).

### Activities and Changes in Services

Free text responses revealed that inpatient palliative care units reported reduced activity; patients who did not have COVID-19 did not want to be admitted for fear of contracting the infection. There was increased home and hospital palliative care team activity. Assistance from volunteers plummeted; of responding services who used volunteers, 79% used them much less. Many volunteers were from older age groups and therefore high risk. Other activities and changes highlighted in free text comments included (Supplementary Tables S1, S3):

• A surge in the number of patients cared for;
• Developing guidelines and education materials as none existed nationally;
• Training and supporting other health professionals;
• Increasing virtual and telephone monitoring (84% of services increased this type of support for patients, and 95% of services in supporting other professionals);
• Directly supporting continuous positive airway pressure or other ventilatory withdrawal;
• Delivering or supporting community or district nursing services;
• Switching from proactive to reactive care because of the demand on services.

**Fig. 1:**
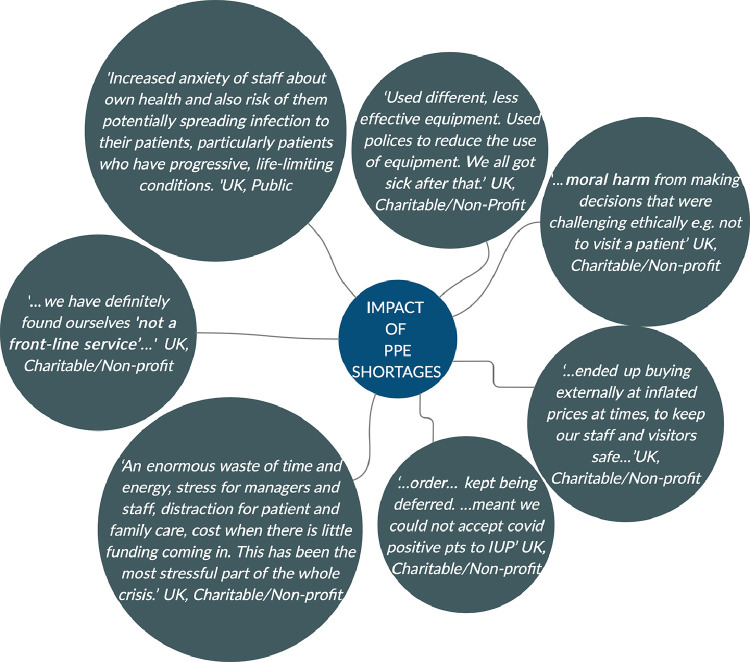
Quotes on the impact of PPE shortages on palliative care services, derived from free text responses.

Free text comments described how staff were stressed by concerns for the patient's health and their own, being unable to visit patients and adapting to new practices. Services were frustrated by how their time and energy were consumed trying to source equipment, including PPE; without which staff got ill or could not deliver care (Fig. 1). The lack of timely PPE and the ethical challenges this presented, in terms of whom they could visit, made them fearful and anxious. There were financial considerations, with some staff especially from charities, concerned for the viability of services (Fig. 1; Supplementary Table S3).

### Shortages and Associated Factors

Of responding services, overall 48% reported shortages of PPE, 40% shortages of staff, 24% shortages of medicines, and 14% shortages of other equipment, commonly battery operated syringe drivers (Table 2).

#### PPE Shortages

**Fig. 2:**
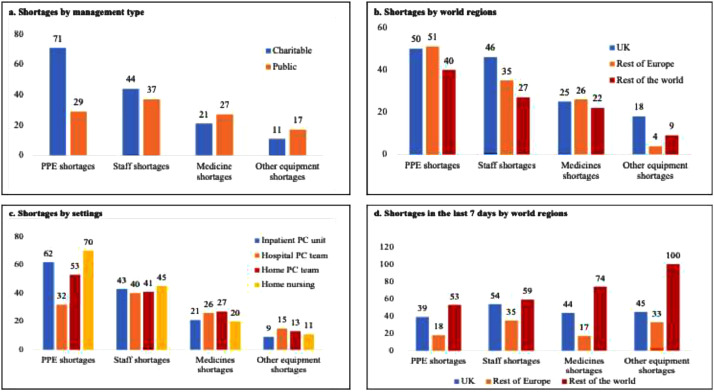
Percentage of shortages by management type, world regions and settings, derived from responses to closed format questions.

Being charity managed was associated with greater likelihood of PPE shortages compared with publicly managed (OR 3.07, 95% CI 1.81–5.20; *P <* .001) (Table 3, Fig. 2). Inpatient palliative care units were more likely to have PPE shortages compared to other settings (OR 2.34, 95% CI 1.46–3.75; *P <* .001). Home nursing had high shortages of PPE (70%, Fig. 2) compared with other settings. Home nursing was most commonly provided by services also providing inpatient palliative care (see supplementary table S2); this may be confounding the relationship between inpatient palliative care units and PPE shortages. Hospital palliative care teams were less likely to have PPE shortages compared to other settings (OR 0.51, 95% CI 0.32–0.82; *P =* .005). During the 7 days before completion of the survey, shortages were more common in the UK than in Europe (Table 2, Fig.2). In free text fields, shortages were reported most commonly for masks (filtering facepiece, FFP2, FFP3), FIT testing kits for FFP3 masks, hospital scrubs, aprons, gloves, face shields, long sleeve gowns, hand gels, goggles, and eye protection.

**Table 3:** Multivariate Logistic Regression: Factors Associated with Shortages

**Multivariate logistic Regression: factors associated with PPE shortages**				
**Variables**	**Odds Ratio**	**CI_lower_**	**CI_upper_**	***P* value**
**Unit management type**				
Public	Ref			
Charitable	3.07	1.81	5.20	P < .001
Other	0.97	0.46	2.05	P = .95
**Inpatient palliative care unit**				
No	Ref			
Yes	2.34	1.46	3.75	P < .001
**Hospital palliative care team**				
No	Ref			
Yes	0.51	0.32	0.82	P = .005
**Multivariate logistic regression: factors associated with staff shortages**				
**Variables**	**Odds ratio**	**CI_lower_**	**CI_upper_**	***P* value**
**Country**				
UK	Ref			
Rest of Europe	0.63	0.37	1.07	.09
Rest of the world	0.44	0.26	0.76	.003
**Multivariate logistic regression: factors associated with other equipment shortages**				
**Variables**	**Odds ratio**	**CI_lower_**	**CI_upper_**	***P* value**
**Inpatient palliative care unit**				
No	Ref			
Yes	0.35	0.18	0.65	.001
**Country**				
UK	Ref			
Rest of Europe	0.15	0.04	0.51	.002
Rest of the world	0.46	0.20	1.08	.07
**Level of busyness**				
About the same	Ref			
A lot more busy	10.81	3.10	37.71	<.001
Slightly more busy	5.41	1.48	19.76	.01
Slightly less busy	3.24	0.81	12.89	.10
Much less busy	1.32	0.21	8.34	.77

Note: No multivariate logistic regression analysis could be carried out for medicines shortages because only one variable (inpatient palliative care unit) was relevant from the univariable analysis.

Ref: Reference category, CI = confidence interval.

#### Staff Shortages

Staff shortages were higher in the UK (46%) than other countries (27%) (Fig. 2, regression analysis OR 0.44, 95% CI 0.26–0.76; *P* = .003, UK vs. rest of the world). Other factors were not significantly associated with staff shortages in regression analysis. Free text comments described shortages in specialist palliative care teams, of doctors (consultants, specialty doctors, middle grade, and junior doctors), nurses (advanced practitioners, clinical nurse specialists, community nurses, community palliative care nurses, registered general nurses, ward managers), allied health professionals (healthcare assistants, occupational therapists, pharmacists, pharmacy assistants, physiotherapists, social workers), administrative and housekeeping staff.

#### Medicines Shortages

The top three medicine shortages reported in free text comments were: levomepromazine, midazolam (used for symptoms of agitation and delirium) and alfentanil (used for pain and breathlessness, commonly used in the UK in severe renal impairment, when morphine is contra-indicated). Shortages of medicines affected 20%–27% of settings (Fig.2). Only one variable (inpatient palliative care unit) met our criteria for inclusion from the univariable analysis (at *P =* .07), so multiple regression analysis was not attempted.

#### Other Equipment Shortages

Free text responses described shortages or delays in access to other equipment, especially syringe drivers/pumps, syringe pump lines, butterfly needles and tympanic thermometer covers. Inpatient palliative care units were less likely to have other equipment shortages compared to other settings (OR 0.35, 95% CI 0.18–0.65; *P = .*001, Fig. 2). When compared to the UK, being elsewhere in Europe was associated with lower odds of other equipment shortages (OR 0.15, 0.04–0.51; *P = .*002).

### Response to Shortages

**Fig. 3:**
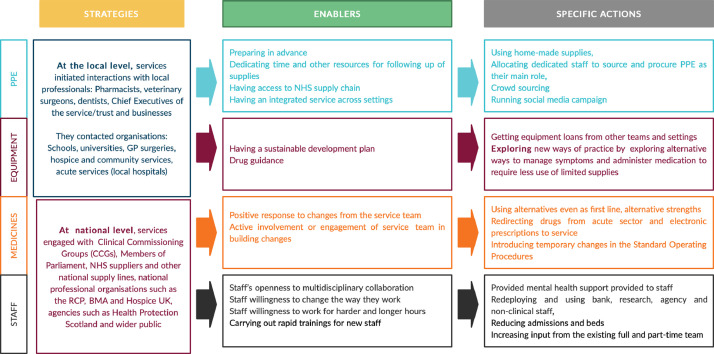
Strategies, enablers, specific actions and quotes about shortages, derived from free text responses.

Free text comments revealed that services expended huge efforts to procure PPE, some deployed staff to find PPE as their main role. Services contacted local vets, schools, dentists, universities, hospitals, businesses, health services, national supply lines, the government, professional organizations and the wider public. Some made their own supplies, crowd sourced and ran social media campaigns (Fig. 3).

In response to staff shortages, multidisciplinary full and part-time staff worked longer hours, extra shifts and flexibly collaborated. Services reduced hospice beds; day-care services were closed and staff were deployed to the community. Bank, research, agency and nonclinical staff were used. New staff were rapidly trained. Some services drafted emergency staffing plans. Mental health support was provided. As one service lead summarized “the impact of the multiple 'threats' to self [that staff felt] should not be underestimated” as the staff are worried about themselves, their families, their colleagues, their patients, working in unfamiliar areas and experiencing “…[Acute Respiratory Disease Syndrome] as a distressing mode of death.”

In response to medicine shortages, services contacted local services and pharmacists (Fig. 3). Some used alternative medicines even as first line, alternative strengths (lower or higher concentrations), or reduced the numbers of vials when prescribing home medications. For example, for shortages of levomepromazine, services prioritized it for agitation, using alternatives for controlling nausea and vomiting. Services introduced temporary changes in Standard Operating Procedures (SOP) and developed new SOPs on drug reuse. The positive response and active involvement of local teams enabled the adaptation, as one service reported ‘…staff [were] very engaged and responsive to our requests for frequent change of practice’.

In response to shortages of other equipment, services tried to get loans from other teams and settings (Fig. 3). The teams explored alternative ways to manage symptoms and administer medication to require less use of limited equipment. For example, faced with a shortage of syringe drivers (small, portable, battery powered infusion pumps suitable to give medicines for breathlessness, pain and agitation), services carried out risk assessments, programmed alternative pumps, such as 50 ml infusion devices, gave four hourly subcutaneous injections, or considered using transdermal patches rather than subcutaneous infusions.

## Discussion

We report the first multinational survey on the response of and challenges to palliative care services during the COVID-19 pandemic. Across all settings palliative care services rapidly changed, increasing the volume and nature of provision, caring for people with COVID-19, as well as for existing patients. Patients dying from and with severe symptoms due to COVID-19 were in three main categories: patients with underlying conditions and/or multimorbid disease not previously known to palliative care, patients already known to palliative care services, and patients previously healthy, who were now dying from COVID-19. Palliative care became exceptionally busy, especially in areas with high COVID-19 prevalence. Care shifted to community and hospital support from inpatient palliative care units. Staff were also infected by COVID-19. Services experienced multiple shortages of equipment, including PPE, staff and medicines that limited their ability to respond. Our two prior null hypotheses were rejected. There were differences in shortages between services with different management types and between settings.

Palliative care staff responded dynamically; they provided care directly to patients and families across hospitals and the community, supported other clinical staff through training, symptom management, communication and care guidance, supported decision making and filled gaps in care. They used their expertise to propose strategies to deal with medicines shortages. These contributions have been vital; clinicians with limited or no palliative care experience had to provide end of life care to patients and support their families.^8^^,^^9^. Palliative care clinicians adapted and innovated quickly.^19^, possibly helped by prior experience with patients with different diseases and with multimorbidity. Palliative care puts the person before their disease wherever they are cared for: it is neither disease nor setting specific. The symptoms and problems of severe COVID-19 are commonly breathlessness and agitation,^20^^,^^21^ both familiar to palliative care clinicians. Current case reports suggest that these symptoms in end stage COVID-19 can be alleviated with low doses of opioids and benzodiazepines, delivered subcutaneously with a battery operated syringe driver.^8^^,^^9^^,^^20^^,^^21^ Palliative care has expertise in holistic end of life care, care for older people and those with multimorbidity.^22^ This flexibility, expertise and learning will be crucial to the international response to COVID-19, especially as cases of COVID-19 continue to rise across the globe.

Our study identified three different groups of patients, and these may require different approaches. A parallel planning approach may be needed for patients with uncertain trajectories, as is often used among patients with other uncertain prognosis in serious illness.^23^^,^^24^ Parallel planning provides for two sets of plans, run side by side. Both plans aim to ensure symptom management and the best in care: one plan is towards improvement or recovery, the second plan is in case the patient deteriorates or begins to die.^24^ Parallel plans may lessen concerns about care rationing and communication, which emerged from public consultation.^25^

Despite efforts to respond and their resilience, palliative care services reported considerable shortages of PPE, staff, medicines and other equipment. PPE shortages especially affected charity managed services. Almost half of services were charity managed; these had lower levels of integration with national health systems than publicly managed services. It is six years since the World Health Assembly resolution in 2014, called for better integration of palliative care into health care systems; a declaration endorsed by all countries.^26^ Our findings reveal little progress. Most countries of the globe lack palliative care doctors, nurses, allied health professionals and trainees to meet their needs.^27^^,^^28^ Undergraduate doctors and nurses receive scant training in palliative care, leaving them lacking in confidence and skills in symptom management, communication and care.^29^^,^^30^ Undergraduate and postgraduate training from expert palliative care clinicians is urgent, as well as developing an adequate palliative care workforce to support services and patients day-to-day, especially as COVID-19 surges continue.

Home nursing and inpatient palliative care units were most seriously affected by PPE shortages. This continued even in the seven days before survey completion, especially in the UK. Many palliative care patients chose not to come to hospital, preferring care at home for fear of contracting COVID-19 and/or wishing to remain close to those important to them, a result supported by research in other pandemics.^12^^,^^31^ and data from the first wave of the COVID-19 pandemic.^32^ Taken together these findings are concerning. If patients are to remain in the community, then the community and care homes, as well as hospitals, need sufficient resources to be able to provide care; this includes Personal Protective Equipment, syringe drivers to deliver subcutaneous infusions to control symptoms and sufficient (and as we moved forward, adequately vaccinated) staff and medicines. Legislation on the reuse of medicines in the community, especially at times of shortages, may need to be revised. The role of free standing inpatient palliative care units during pandemics is worthy of consideration and planning: could their staff proactively be diverted to the community (as occurred in many settings in our study), could they be diverted to hospital palliative care teams or care homes (both settings needing additional support), or could they provide an alternative or rehabilitation/step down care from hospitals? Any option would need planning, training, and possibly a different skill mix.

## Limitations and Strengths

The findings of this study are limited by its cross-sectional design; causal relationships cannot be confirmed. There may be nonresponse, sample and other biases. We do not know whether services who did not respond had different experiences, with more or fewer challenges. The survey was distributed through networks known to us, mainly UK and European. (Although approached, some other countries, e.g., Australia, were unable to distribute the survey to palliative care networks.) The survey was offered only in the English language; most responses were from the UK. Some countries were not represented, or had few responses. Country level effects and clustering may be important, we did not have sufficient data to explore this. At the time of the survey the pandemic was affecting regions differently, which might have affected responses. These limit the interpretation of our international comparisons. Responses may have been influenced by social desirability. Free-text comments are a useful source of information,.^33^, but may not represent all respondents. Nevertheless, this is the first study to provide systematic multinational data on the palliative care response and challenges faced during the COVID-19 pandemic. We used a combination of networks to try to obtain a wide response; there is no comprehensive multinational network of palliative care services from which we could sample. We combined closed and free text responses to generate in depth findings that supported, shed further light on and complemented the findings from closed responses, providing a more robust, detailed and inclusive response than earlier surveys.

## Conclusion

Palliative care services responded actively but most felt ignored by national health systems during the COVID-19 pandemic, despite supporting patients who were dying or had severe symptoms, supporting their families/carers, and supporting other professionals to deliver care. Services provided expertise in symptom management and holistic care while facing shortages of equipment, staff and medicines. The crucial role of palliative care during pandemics must be better recognized and integrated. This is particularly the case for charity managed services and those providing care in people's homes. Beyond COVID-19, this research has shed light on the limited integration of palliative care, the urgent need to increase its workforce and a need for palliative skills to be a core part of the training of clinicians.

## Copyright

The CovPall Questionnaire© is copyright. License for use of the questionnaire is granted free of charge. However, you may not charge for use of the questionnaire. All reproductions of the questionnaire should contain the statement: “Reproduced with the permission of Irene J Higginson, Cicely Saunders Institute of Palliative Care, Policy and Rehabilitation, King's College London, on behalf of the CovPall Study Team, as the Chief Investigator of CovPall study and the Intellectual Property owner of the CovPall Survey”.

In all publications, presentations and when the name of the survey is used, relevant CovPall papers should be referenced, and permission for use of the survey should be acknowledged.

## Data Sharing

Applications for use of the survey data can be made for up to 10 years, and will be considered on a case by case basis on receipt of a methodological sound proposal to achieve aims in line with the original protocol. The study protocol is available on request. All requests for data access should be addressed to the Chief Investigator via the details on the CovPall website (https://www.kcl.ac.uk/cicelysaunders/research/evaluating/covpall-study, and palliativecare@kcl.ac.uk) and will be reviewed by the Study Steering Group.

## Conflict of Interest

IJH is a National Institute for Health Research (NIHR) Emeritus Senior Investigator and is supported by the NIHR Applied Research Collaboration (ARC) South London (SL) at King's College Hospital National Health Service Foundation Trust. IJH leads the Palliative and End of Life Care theme of the NIHR ARC SL and co-leads the national theme in this. MM is funded by a NIHR Career Development Fellowship (CDF-2017-10-009) and NIHR ARC SL. LF is funded by a NIHR Career Development Fellowship (CDF-2018-11-ST2-002). KS is funded by a NIHR Clinician Scientist Fellowship (CS-2015-15-005). RC is funded by Cicely Saunders International. FEM is a NIHR Senior Investigator. MBH is supported by the NIHR ARC SL. The views expressed in this article are those of the authors and not necessarily those of the NIHR, or the Department of Health and Social Care.

## Appendix Supplementary materials

Image, application 1

Image, application 2

Image, application 3

Image, application 4
