# Supplementary material for: The Challenges of Caring for People Dying From COVID-19: A Multinational, Observational Study (CovPall)
Source: J Pain Symptom Manage. 2021 Sep;62(3):460–70. doi: 10.1016/j.jpainsymman.2021.01.138 (PMC7863772; doi:10.1016/j.jpainsymman.2021.01.138)
Supplement: Supplementary file 1 [file mmc1.docx]

**Checklist of MORECare Statement**

| Category | Checklist items |   Answer |
| --- | --- | --- |
| Introduction/background | 1. Present theoretical framework for the intervention and levels of need established |   Not applicable |
|  | 2. Present objectives appropriate to the level of intervention development |   Not applicable  |
| Study design   | 3. Indicate and justify stage in MRC guidance for development and evaluation of complex interventions, for example, feasibility, preliminary evaluation, efficacy/cost effectiveness and wider effectiveness | Not applicable |
|  | 4. Feasibility stages should test both feasibility of the intervention and of methods of evaluation, including outcome measurement |   Not applicable |
|  | 5. Justify methods, considering appropriate use of existing data sets and secondary analysis as these may produce rapid information | Yes  Page 5 |
|  | 6. Justify methods of empirical studies considering mixed methods, observational studies and randomised trials |   Yes  Pages 5   |
| Study team | 7. Ensure involvement from: (i) consumers, patients and caregivers; (ii) relevant clinicians; (iii) relevant methodologists to develop study questions, questionnaires and procedures; and (iv) researchers familiar with the challenges in EoLC studies | Yes. This research developed in response to a recent consultation with our existing patient and public involvement and engagement (PPIE) networks. Our study team and steering group includes clinicians and experts in the palliative care. |
|  | 8. Ideally, involvement should be well established and continuing, beyond a specific study, with joint meetings or rotations between clinical and research staff | We have weekly CovPall Team meetings that include clinical and research staff. We also have wider meetings and engagement with services through Hospice UK ECHO meetings, where we attend and regularly present findings. |
|   Ethics   | 9. Note in ethics committee application MORECare recommendations that it is ethically desirable for patients and families in EoLC to be offered involvement in research and MORECare evidence of patient willingness to be approached | Participants surveyed about their services were palliative care service leads. The research proposal developed in response to a recent consultation with our existing patient and public involvement and engagement (PPIE) networks. We received >40 responses via telephone, email and our online forum (www.csipublicinvolvement.co.uk). These identified the challenges for patients, their families, and members of the public, in relation to palliative and end of life care during the COVID-19 outbreak. |
|  |   10. Work within legal frameworks on mental capacity, consent and so on, to ensure that those who may benefit from interventions are offered an opportunity to participate if they wish | Not applicable |
|  | 11. Collaborate with patients and caregivers in the design of the study, vocabulary used in explaining the study, consent procedures and any ethical aspects | The research proposal developed in response to a recent consultation with our existing patient and public involvement and engagement (PPIE) networks. We received >40 responses via telephone, email and our online forum (www.csipublicinvolvement.co.uk). These identified the challenges for patients, their families, and members of the public, in relation to palliative and end of life care during the COVID-19 outbreak. Two patient and public involvement (PPI) members are part of our steering group. |
|  | 12. Attend the ethics committee meeting with a caregiver or patient, as a means to help the committee better understand the patient perspective |   We did not attend the ethics committee meeting with a caregiver or patient. However, patients and caregivers perspectives informed this research. |
|  | 13. Ensure proportionality in patient and caregiver information sheets, appropriate to the study design and level of risk, as excessive information in itself can be tiring/distressing for very ill individuals | Not applicable |
| Participants | 14. Adjust eligibility criteria to recruit those patients who may benefit most from intervention, ensuring equipoise | Not applicable |
| Procedures | 15. Minimise burden for existing clinical staff for participation in the study | We minimised the burden of participation in this survey for palliative care leads. The palliative care providers were allowed to choose how they would like to provide data to minimise the burden to them. They had three options:  (a) to themselves enter data directly online into the bespoke data base, at a time of their choosing,  (b) if they preferred, they could give the information to a trained interviewer over the telephone or virtual connection (e.g. via Microsoft teams or Zoom) who entered the data for them, or  (c) if they preferred, be sent the survey as a word document via email to complete and return electronically (e.g. via secure NHS email).  There was flexibility around data entry with palliative care providers being able to enter data within 14-weeks. |
|  | 16. Clearly distinguish between service received and research activity interviews in study arms when multiple interviews with patients are undertaken in trials, for example, using a graphical system | Not applicable   |
| Outcome measures   | 17. Choose outcome measures that meet the following criteria:   - established validity and reliability in relevant population - responsive to change over time - capture clinically important data - easy to administer and interpret (for example, short and with low level of complexity) - applicable across care settings to capture change in outcomes by location (for example, patients’ home, hospital, hospice) - able to be integrated into clinical care - minimise problems of response shift | Not applicable to survey |
|  | 18. Consider including patients’ experience of care, as this is central to many interventions |   Not applicable |
|  |   19. Select time points of outcome measurement to balance the value of early recording, to reduce attrition, but to allow enough time for the intervention to have had an effect | Not applicable |
|  | 20. Consider the potential effect of response shift (that is, a change in a person’s internal conceptualisation or calibration of the aspects measured). Questionnaires that include anchor points or descriptions of each response category may be less problematic in this regard | This has been considered and anchor time points and descriptions of response category are provided. |
| Missing data and attrition considerations | 21. Estimate in advance levels of, and reasons for, attrition and missing data, integrating these into sample size estimates and planned collection of data from proxies | Yes – page 6  After removing duplicate entries and ineligible entries (blank entries or where individuals indicated they were not from a palliative care service) all available data during the study period were analysed. Missing data were not imputed due to limitations inherent in the commonly used approaches for handling missing data. |
|  | 22. Monitor during the study and report all levels of, and reasons for, attrition and other missing data | Yes – page 6  We could not report reasons for missing data in this survey. |
|  | 23. Assume missing quantitative data NOT to be at random unless proven otherwise | Given the small number of missing data, we did not explore whether data were missing at random or not. Furthermore, missing data were not imputed due to limitations inherent in the commonly used approaches for handling missing data. |
|  | 24. Test results from different methods of imputation – noting that ‘using only complete cases’ is a form of imputation | We used only complete cases in the analysis.   |
|  | 25. Use the MORECARE classification of attrition to describe causes of attrition: that is,   - ADD – attrition due to death; - ADI - attrition due to illness;   AaR - attrition at random. | Not applicable |
|  |   26. Consider reasons for missing data which are not due to attrition, for example missed questionnaire, or missed data item in questionnaire. Consider these in analysis and the potential imputations | While it is possible that some of survey respondents missed data items on the questionnaire, we did not explore this. We acknowledged the presence of missing data as a study limitation. |
|   Mixed method studies   |   27. Mixed methods can be appropriate in all phases of development and evaluation | This study was not a mixed methods study. It was a multinational online survey of clinical leads of palliative care services. Free-text explanatory comments were also invited. |
|  |   28. Ensure appropriate multi-disciplinary skills mix or training of team   | The CovPall team is multidisciplinary including quantitative and qualitative researchers, mixed methods researchers as well as people with different clinical backgrounds. The team includes doctors, nurses, a physiotherapist, and a pharmacist. |
|  | 29. Define the theoretical paradigm and method of integrating results and safeguards to ensure rigour at the outset | Not applicable |
|  | 30. Plan investigation to avoid undue burden of qualitative and quantitative questionnaires – perhaps dividing data collection or selecting questions and/or sampling appropriately   | Not applicable |
|  | 31. Take into account any potential therapeutic effect of qualitative interviews where participants can express their feelings, if these are similar to components of the intervention |   Not applicable |
|  | 32. Ensure that those collecting data are appropriately trained in qualitative data collection | Not applicable |
| Implementation | 33. Consider implementation implications, including workforce and training needs, in all phases of the study | Not applicable |
| Cost-effectiveness   | 34. Integrate into preliminary evaluations and test feasibility of methods |   Not applicable |
|  | 35. Collect data on use of services including health, voluntary, social and informal care, to take societal approach to care costs | Not applicable |
|  | 36. Justify appropriate outcome measures to generate cost effectiveness | Not applicable   |
