# Supplementary material for: The Challenges of Caring for People Dying From COVID-19: A Multinational, Observational Study (CovPall)
Source: J Pain Symptom Manage. 2021 Sep;62(3):460–70. doi: 10.1016/j.jpainsymman.2021.01.138 (PMC7863772; doi:10.1016/j.jpainsymman.2021.01.138)
Supplement: Supplementary file 2 [file mmc2.docx]

**CovPall survey, checklist for Reporting Results of Internet E-Surveys (CHERRIES)**

| 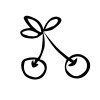 | **Checklist for Reporting Results of Internet E-Surveys (CHERRIES)** | | |
| --- | --- | --- | --- |
| ***Item Category*** | ***Checklist Item*** | ***Description*** | ***PAGE*** |
| **Design** |  |  |  |
|  | Describe survey design | Services were identified and contacted through national and multinational palliative care and hospice organisations and provided with a link to complete an on-line survey. | 5 |
| **IRB (Institutional Review Board) approval and informed consent process** |  |  |  |
|  | IRB approval | The survey received ethical (Institutional Review Board) approval from King’s College London Research Ethics committee (LRS-19/20-18541) | 5 |
|  | Informed consent | Services were identified and contacted through national and multinational palliative care and hospice organisations and provided with a link to an on-line survey to be completed by the medical or nursing lead or their nominee. Completion indicated consent. The email attached the participant information sheet with details of the study rationale, ethical approval, data protection and management, investigators and contacts for further information or concern. It was intended to be brief, taking around 30 minutes to complete. Free-text explanatory comments were invited. | 5 |
|  | Data protection | We developed and piloted a secure, password-protected web-based data entry portal (in the Research Electronic Data Capture (REDCap) at the study coordinating centre, King’s College London. Services could keep their identity hidden if they wished, but most chose to provide an email for contact. Data were anonymised before analysis. | 5, Supplementary file Box S1 |
| **Development and pre-testing** |  |  |  |
|  | Development and testing | The questionnaire was developed and piloted by the CovPall study team building on an earlier survey of Italian hospices, adding questions on the impact of and response to COVID-19. The study team developed and piloted a secure, password-protected web-based data entry portal (in the Research Electronic Data Capture (REDCap) at the study coordinating centre, King’s College London. | 5, Supplementary file Box S1 |
| **Recruitment process and description of the sample having access to the questionnaire** |  |  |  |
|  | Open survey versus closed survey | This was a closed survey. Services were identified and contacted through national and multinational gatekeeper palliative care and hospice organisations and asked that their medical or nursing lead, or their nominee, complete an on-line survey available via a link. The study team developed and piloted a secure, password-protected web-based data entry portal (in the Research Electronic Data Capture (REDCap) at the study coordinating centre, King’s College London. | 5, Supplementary file Box S1 |
|  | Contact mode | Services were identified and contacted through national and multinational gatekeeper palliative care and hospice organisations | 5, Supplementary file Box S1 |
|  | Advertising the survey | Services were identified and contacted through national and multinational gatekeeper palliative care and hospice organisations. The email attached the participant information sheet with details of the study rationale, ethical approval, data protection and management, investigators and contacts for further information or concern. No incentives were offered for completion. The CovPall study was presented at relevant on-line meetings and discussed with gatekeeper organisations and in blogs, to inform the methods, questions and to raise awareness and engagement. | 5, Supplementary file Box S1 |
| **Survey administration** |  |  |  |
|  | Web/E-mail | Services were identified and contacted through national and multinational gatekeeper palliative care and hospice organisations and asked that their medical or nursing lead, or their nominee, complete an on-line survey available via a link. The study team developed and piloted a secure, password-protected web-based data entry portal (in the Research Electronic Data Capture (REDCap) at the study coordinating centre, King’s College London. | 5, Supplementary file Box S1 |
|  | Context | As stated above, services were identified and contacted through national and multinational gatekeeper palliative care and hospice organisations and asked that their medical or nursing lead, or their nominee, complete an on-line survey available via a link. | 5, Supplementary file Box S1 |
|  | Mandatory/voluntary | It was a voluntary survey as service leads were not mandated to complete it. | Supplementary file, appendix II |
|  | Incentives | There was no incentive offered for survey completion | Supplementary file Box S1 |
|  | Time/Date | The survey opened on April 23rd and closed July 31st 2020 | Supplementary file Box S1 |
|  | Randomization of items or questionnaires | Items were not randomised or alternated. | Supplementary file, appendix II |
|  | Adaptive questioning | We used adaptive questioning in order to reduce the number and complexity of questions. | Supplementary file, appendix II |
|  | Number of Items | We had an average of 12 questionnaire items per page | Supplementary file, appendix II |
|  | Number of screens (pages) | 16 pages | Supplementary file, appendix II |
|  | Completeness check | The research team audited the data weekly to ensure data entry completeness and sent monthly missing data and incomplete entry reports to the research associates and administrators, and where consent permitted to relevant respondents to check validity. | Supplementary file Box S1 |
|  | Review step | Respondents could review and change their answers before submitting the survey. They could also pause the questionnaire by clicking the “Save and Return Later” button at the bottom of each page. There were given a code to enable them continue later. There was a “returning” option at the top right of the page. If respondents wanted to correct any errors after submitting the survey, they were informed that they could email the research team at [palliativecare@kcl.ac.uk](mailto:palliativecare@kcl.ac.uk) with CovPall in the subject line | Supplementary file, appendix II |
| **Response rates** |  |  |  |
|  | Unique site visitor | In total 489 questionnaire were commenced, 477 completed (completion rate 97·5%); of these 15 were duplicates and 2 triplicates of entries with the same name/email; 2 were invalid being from one researcher without a palliative care service, leaving 458 valid responses. We were able to determine this through the email addresses provided. | 6 |
|  | View rate (Ratio of unique survey visitors/unique site visitors) | Not applicable. Respondents were invited to participate through national and multinational gatekeeper palliative care and hospice organisations | 5 |
|  | Participation rate (Ratio of unique visitors who agreed to participate/unique first survey page visitors) | Not applicable. Respondents were invited to participate through national and multinational gatekeeper palliative care and hospice organisations | 5 |
|  | Completion rate (Ratio of users who finished the survey/users who agreed to participate) | In total 489 questionnaire were commenced, 477 completed (completion rate 97·5%); of these 15 were duplicates and 2 triplicates of entries with the same name/email; 2 were invalid being from one researcher without a palliative care service, leaving 458 valid responses. | 6 |
| **Preventing multiple entries from the same individual** |  |  |  |
|  | Cookies used | Cookies were not used. In total 489 questionnaire were commenced, 477 completed (completion rate 97·5%); of these 15 were duplicates and 2 triplicates of entries with the same name/email; 2 were invalid being from one researcher without a palliative care service, leaving 458 valid responses | 6 |
|  | IP check | IP check was not carried out. However, as stated above, the unique email addresses were used to identify duplicate entries. Respondents with duplicate entries were contacted and asked which entry should be included in the analysis. | 6 |
|  | Log file analysis | As stated above, the unique email addresses were used to identify duplicate entries. Respondents with duplicate entries were contacted and asked which entry should be included in the analysis. | 6 |
|  | Registration | As stated above, the unique email addresses were used to identify duplicate entries. Respondents with duplicate entries were contacted and asked which entry should be included in the analysis. | 6 |
| **Analysis** |  |  |  |
|  | Handling of incomplete questionnaires | After removing duplicates and ineligible entries all available data were analysed. Missing data were not imputed. | 6 |
|  | Questionnaires submitted with an atypical timestamp | Not applicable. After removing duplicates and ineligible entries all available data were analysed. | 6 |
|  | Statistical correction | Not applicable |  |
