# Supplementary material for: The Challenges of Caring for People Dying From COVID-19: A Multinational, Observational Study (CovPall)
Source: J Pain Symptom Manage. 2021 Sep;62(3):460–70. doi: 10.1016/j.jpainsymman.2021.01.138 (PMC7863772; doi:10.1016/j.jpainsymman.2021.01.138)
Supplement: Supplementary file 3 [file mmc3.pdf]

Supplementary Material

Appendix I

CovPall

Box S 1 Procedures for CovPall survey

Services were identified and contacted through national and multinational gatekeeper palliative care and hospice organisations (see acknowledgements and supplementary appendix III for details) and asked that their medical or nursing lead, or their nominee, complete an on-line survey available via a link. The email attached the participant information sheet with details of the study rationale, ethical approval, data protection and management, investigators and contacts for further information or concern. No incentives were offered for completion. Completion was taken to indicate informed consent. The CovPall study was presented at relevant on-line meetings and discussed with gatekeeper organisations and in blogs, to inform the methods, questions and to raise awareness and engagement.

We developed and piloted a secure, password-protected web-based data entry portal (in the Research Electronic Data Capture (REDCap) at the study coordinating centre, King's College London. Services could keep their identity hidden if they wished, but most chose to provide an email for contact. Data were anonymised before analysis.

The questionnaire was developed and piloted by the CovPall study team building on an earlier survey of Italian hospices, adding questions on the impact of and response to COVID-19. It was intended to be brief, taking around 30 minutes to complete as we recognised staff were busy, and comprised seven sections: region and responder; Palliative Care (PC)/hospice management and services offered pre-COVID-19; experiences of patients and staff with COVID-19; changes to services overall; changes in specific settings; shortages experienced; symptom management. Free-text explanatory comments were invited in all sections and respondents also indicated whether and how they could be contacted. Respondents could save and complete the questionnaire later if they wished. The questionnaire is available in supplementary appendix II.

The survey opened on April 23rd and closed July 31st 2020. The study coordinating centre replied actively to respondents queries or requests for help.

In most cases, data collection and entry was done electronically via the REDCap link directly by the services without problems, however a few palliative care services had difficulties accessing the online survey due to local

computer procedures. In these instances they were sent the survey in paper format, completed this and returned it to King's to enter this data, or were interviewed over the phone and data entry sheet was completed by the researchers. The research team also audited the data weekly to ensure data entry completeness and sent monthly missing data and incomplete entry reports to the research associates and administrators, and where consent permitted to relevant respondents to check validity.

Table S 1 Characteristics of responding palliative care and hospice services by region

	**UK**	**Rest of Europe**	**Rest of the world (n = 95)**			**Total**
	**(n = 277)**	**(n = 85)**	**LIC/LMIC (n = 17)**	**UMIC (n = 19)**	**HIC (n = 59)**	
**Role of respondents** (n/N, %)[a]						
Medical director/ lead medical clinician	97/274 (35.4%)	42/85 (49.4%)	11/17 (64.7%)	7/19 (36.8%)	26/58 (44.8%)	183/453 (40.4%)
Nurse director/ lead nurse clinician	69/274 (25.2%)	8/85 (9.4%)	2/17 (11.8%)	2/19 (10.5%)	7/58 (12.1%)	88/453 (19.4%)
Other	108/274 (39.4%)	35/85 (41.2%)	4/17 (23.5%)	10/19 (52.6%)	25/58 (43.1%)	182/453 (40.2%)
Missing	3	-	-	-	1	5[b]
**Management type** (n/N, %)[a]						
Charitable / non-profit	143/262 (54.6%)	23/85 (27.1%)	5/17 (29.4%)	6/17 (35.3%)	14/58 (24.1%)	192/440 (43.6%)[c]
Public	103/262 (39.3%)	51/85 (60%)	8/17 (47.1%)	6/17 (35.3%)	36/58 (62.1%)	204/440 (46.4%)[b]
Private	1/262 (0.4%)	9/85 (10.6%)	2/17 (11.8%)	5/17 (29.4%)	2/58(3.4 %)	19/440 (4.3%)[b]
Other	15/262 (5.7%)	2/85 (2.4%)	2/17 (11.8%)	0/17 (0%)	6/58 (10.3%)	25/440 (5.7%)[b]
Missing	15	-	-	2	1	18
**Type of service** (n/N, %)						
Adult only	247/277 (89.2%)	68/85 (80%)	7/17 (41.2%)	9/19 (47.4%)	40/59 (67.8%)	371/458 (81%)[b]
Children only	16/277 (5.8%)	5/85 (5.9%)	0/17 (0%)	3/19 (15.8%)	5/59 (8.5%)	29/458 (6.3%)[b]
Both adult and children	11/277 (4%)	10/85 (11.8%)	9/17 (52.9%)	7/19 (36.8%)	14/59 (23.7%)	52/458 (11.4%)[c]

None indicated	3/277 (1.1%)	2/85 (2.4%)	1/17 (5.9%)	0/19 (0%)	0/59 (0%)	6/458 (1.3%)[b]
**Setting** (n/N, %)						
Inpatient PC unit	168/277 (60.6%)	44/85 (51.8%)	8/17 (47.1%)	6/19 (31.6%)	34/59 (57.6%)	261/458 (57%)[c]
Hospital PC team	135/277 (48.7%)	26/85 (30.6%)	9/17 (52.9%)	10/19 (52.6%)	37/59 (62.7%)	217/458 (47.4%)[b]
Home PC team	160/277 (57.8%)	47/85 (55.3%)	10/17 (58.8%)	9/19 (47.4%)	34/59 (57.6%)	261/458 (57%)[c]
Home nursing	92/277 (33.2%)	15/85 (17.6%)	1/17 (5.9%)	4/19 (21.1%)	7/59 (11.9%)	119/458 (26%)[b]
Total	277	85	17	19	59	458[b]
**Setting** (n/N, %)						
None indicated	3/277 (1.1%)	1/85 (1.2%)	1/17 (5.9%)	0/19 (0%)	0/59 (0%)	5/458 (1.1%)[b]
Single setting	110/277 (39.7%)	49/85 (57.6%)	8/17 (47.1%)	11/19 (57.9%)	24/59 (40.7%)	202/458 (44.1%)[b]
Multiple settings (2 or more settings	164/277 (59.2%)	35/85 (41.2%)	8/17 (47.1)	8/19 (42.1%)	35/59 (59.3%)	251/458 (54.8%)[c]
**% funding usually from NHS**						
Mean (SD)	28.3 (13)	41.4 (35)	20 (34.6)	24.6 (42.6)	34.1 (35.3)	30.1 (20.9)
Median (IQR)	25 (20, 35.5)	31.5 (9.8, 70)	0 (0, 50)	10 (0, 56.5)	40 (0, 60)	25 (19, 40)
Total	137	22	5	5	11	181[b]
Missing	140	63	12	14	48	277
**Level of Integration with NHS**						
Mean (SD)	74 (16.8)	72.1 (22.7)	55.4 (31.8)	51.9 (27.5)	68.3 (25.3)	71.5 (21)
Median (IQR)	76 (63.8, 87)	76.5 (66.3, 87)	59 (28.3, 78.5)	49 (29, 77)	73 (49.8, 90)	75 (60.8, 87)

Total<br>Missing	250<br>27	80<br>5	16<br>1	15<br>4	52<br>7	414[b]<br>44
**Information about services offered before COVID-19 pandemic**						
**Bereavement services** (n/N, %)[a]<br>Missing	201/265 (75.8%)<br>12	58/83 (69.9%)<br>2	13/17 (76.5%)<br>-	12/17 (70.6%)<br>2	42/58 (72.4%)<br>1	327/441 (74.1%)[c]<br>17
**Bereavement services offered only to families/friends of patients who had been cared for by your service** (n/N, %)[a]<br>Missing	135/199 (67.8%)<br>78	49/58 (84.5%)<br>27	11/13 (84.6%)<br>4	9/12 (75%)<br>7	26/42 (61.9%)<br>17	231/325 (71.1%)[c]<br>133
**Use of a risk assessment tool to help you decide how to target bereavement services** (n/N, %)[a]<br>Missing	82/189 (43.4%)<br>88	10/57 (17.5%)<br>28	4/13 (30.8%)<br>4	6/12 (50%)<br>7	13/42 (31%)<br>17	115/314 (36.6%)[b]<br>144
**Volunteer roles within service**<br>Direct patient/family facing support (e.g. befriending, home visits, in-patient unit care, family support groups/visiting etc) (n/N, %)	166/277 (59.9%)	44/85 (51.8%)	13/17 (76.5%)	15/19 (78.9%)	30/59 (50.8%)	269/458 (58.7%)[c]
**Volunteer roles within service**<br>Indirect patient/family facing support (e.g. reception functions, refreshments, driving/transport etc) (n/N, %)	164/277 (59.2%)	31/85 (36.5%)	5/17 (29.4%)	7/19 (36.8%)	30/59 (50.8%)	238/458 (52%)[c]

**Volunteer roles within service** Back office functions (e.g. finance support, maintenance, gardening etc) (n/N, %)	162/277 (58.5%)	28/85 (32.9%)	5/17 (29.4%)	5/19 (26.3%)	21/59 (35.6%)	222/458 (48.5%)[c]
**Volunteer roles within service** Fundraising functions (e.g. shop volunteers, lottery etc) (n/N, %)	153/277 (55.2%)	26/85 (30.6%)	3/17 (17.6%)	4/19 (21.1%)	22/59 (37.3%)	209/458 (45.6%)[c]
**Volunteer roles within service** Other (n/N, %)	34/277 (12.3%)	5/85 (5.9%)	0/17 (0%)	3/19 (15.8%)	9/59 (15.3%)	51/458 (11.1%)[b]
**Use of remote consultations to help support patient care or education before COVID-19** Telephone support for education (n/N, %)	56/277 (20.2%)	29/85 (34.1%)	7/17 (41.2%)	9/19 (47.4%)	24/59 (40.7%)	125/458 (27.3%)[b]
**Use of remote consultations to help support patient care or education before COVID-19** Telephone support for clinical care (n/N, %)	179/277 (64.6%)	54/85 (63.5%)	13/17 (76.5%)	11/19 (57.9%)	45/59 (76.3%)	303/458 (66.2%)[c]
**Use of remote consultations to help support patient care or education before COVID-19**	88/277 (31.8%)	11/85 (12.9%)	1/17 (5.9%)	6/19 (31.6%)	21/59 (35.6%)	127/458 (27.7%)[b]

Telehealth/video support/e-learning for education (n/N, %)						
**Use of remote consultations to help support patient care or education before COVID-19**<br>Telehealth/ video support/e-learning for clinical care (n/N, %)	54/277 (19.5%)	11/85 (12.9%)	4/17 (23.5%)	4/19 (21.1%)	26/59 (44.1%)	99/458 (21.6%)[b]
**Number of beds in in-patient hospice/palliative care unit**						
Mean (SD)	16.3 (9.3)	20.5 (31.5)	138.4 (348.5)	20.7 (17.7)	45.5 (171.8)	24.6 (87.8)
Median (IQR)	14.5 (10, 20)	10.5 (8, 19.5)	11 (6.3, 42.5)	14.5 (12.3, 27.5)	11 (8, 20.5)	14 (10, 20)
Total	166	44	8	6	33	258[b]
Missing	111	41	9	13	26	200
**Approximate number of new patients seen annually in in-patient palliative care unit**						
Mean (SD)	348.5 (292.1)	265.6 (257.1)	2756.9 (6970)	185.5 (288.8)	245.6 (197.9)	397.8 (1313)
Median (IQR)	300 (200, 400)	200 (112.5, 328)	395 (87.5, 500)	50 (38.7, 400)	200 (80, 350)	285 (177, 400)
Total	146	44	8	5	31	235[b]
Missing	131	41	9	14	28	223

**Approximate number of new patients seen annually by hospital palliative care team**						
Mean (SD)	1131 (950.4)	504.4 (419.9)	2228 (3884)	268.5 (292)	784.1 (2003.6)	995.1 (1415.7)
Median (IQR)	950 (470, 1677.5)	475 (97.5, 812.5)	300 (31, 4455)	165 (72.5, 425)	300 (100, 700)	700 (200, 1200)
Total	120	26	9	10	35	200
Missing	157	59	8	9	24	258[b]
**Hospital palliative care team supported** (n/N, %)						
Acute hospital	127/135 (94.1%)	26/26 (100%)	7/9 (77.8%)	9/10 (90%)	30/37 (81.1%)	199/217 (91.7%)
Community hospital	34/135 (25.2%)	8/26 (30.8%)	4/9 (44.4%)	3/10 (30%)	15/37 (40.5%)	64/217 (29.5%)
**Hospital palliative care team offered 24 hour support for patients** (n/N, %)	101/135 (74.8%)	12/26 (46.2%)	7/9 (77.8%)	8/10 (80%)	28/37 (75.7%)	156/217 (71.9%)
**Approximate number of new patients seen annually by home palliative care team**						
Mean (SD)	947.5 (756.9)	454 (576.6)	748 (1618.4)	456.3 (573.5)	489.4 (949.4)	761.6 (819.8)
Median (IQR)	775 (363, 1400)	300 (100, 550)	100 (50, 612.5)	175 (62.5, 750)	175 (63.8, 400)	545 (170, 1062.3)
Total	139	47	9	8	32	236[b]
Missing	138	38	8	11	27	222

**Home palliative care team offered support to patients in care homes** (n/N, %)	138/148 (93.2%)	42/47 (89.4%)	6/10 (60%)	7/8 (87.5%)	32/33 (97%)	226/247 (91.5%)[c]
**Home palliative care team offered 24 hour support for patients** (n/N, %)	103/160 (64.4%)	32/47 (68.1%)	7/10 (70%)	9/9 (100%)	23/34 (67.6%)	174/261 (66.7%)[b]
**Approximate number of new patients seen annually by home care**						
Mean (SD)	430.9 (555.3)	99.3 (122.6)	90	450 (312.2)	195.8 (129)	362.8 (500.8)
Median (IQR)	300 (164, 436.5)	50 (30, 120)	90 (90, 90)	350 (200, -)	200 (55, 331.3)	250 (90, 381.3)
Total	73	15	1	3	6	98
Missing	204	70	16	16	53	360[b]
**Home nursing service offered 24 hour support for patients** (n/N, %)	54/92 (58.7%)	6/15 (40%)	0/1 (0%)	3/4 (75%)	4/7 (57.1%)	67/119 (56.3%)
**Experience with suspected or confirmed COVID-19**						
**Confirmed (by test) cases of COVID-19** (n/N, %)[a]						
Yes	228/264 (86.4%)	50/83 (60.2%)	5/16 (31.3%)	3/17 (17.6%)	18/58 (31%)	304/439 (69.2%)[b]
Missing	13	2	1	2	1	19
**Approximate number of confirmed COVID-19 cases**						
Mean (SD)	40.2 (59.5)	23 (35.7)	36.6 (63.5)	9 (9.6)	6.9 (7.7)	35 (54.7)
Median (IQR)	14 (5, 52)	9 (2.5, 28.5)	10 (6.5, 80)	5 (2, -)	4 (1, 11)	10 (4, 50)
Total	215	49	5	3	17	289

Missing	62	36	12	16	42	169[b]
**Services with confirmed COVID-19 cases** (n/N, %)						
In-patient PC unit	132/158 (83.5%)	26/43 (60.5%)	3/8 (37.5%)	1/5 (20%)	10/34 (29.4%)	172/249 (69.1%)[b]
Hospital PC team	117/129 (90.7%)	16/26 (61.5%)	3/9 (33.3%)	2/9 (22.2%)	15/36 (41.7%)	153/209 (73.2%)
Home PC team	135/151 (89.4%)	29/46 (63%)	3/9 (33.3%)	1/7 (14.3%)	9/34 (26.5%)	177/248 (71.4%)[b]
Home nursing	74/87 (85.1%)	7/13 (53.8%)	0/1 (0%)	0/3 (0%)	3/7 (42.9%)	84/111 (75.7%)
**Suspected cases of COVID-19 (untested but clinical diagnosis/symptoms)** (n/N, %)[a]	208/260 (80%)	46/83 (55.4%)	7/17 (41.2%)	6/17 (35.3%)	27/58 (46.6%)	295/436 (67.7%)[c]
Missing	17	2	-	2	1	22
**Approximate number of suspected COVID-19 cases**						
Mean (SD)	22.9 (42.2)	16.4 (30.4)	20.4 (35.9)	7.7 (7.1)	8.3 (10.4)	20 (38)
Median (IQR)	10 (4, 21)	10 (5, 16)	3 (1, 20)	7 (1, 12.5)	5 (2, 10)	10 (4, 20)
Total	193	47	7	6	26	280[b]
Missing	84	38	10	13	33	178
**Services with suspected COVID-19 cases** (n/N, %)						
In-patient PC unit	122/156 (78.2%)	21/43 (48.8%)	2/8 (25%)	3/5 (60%)	16/34 (47.1%)	165/247 (66.8%)[c]
Hospital PC team	105/125 (84%)	16/26 (61.5%)	4/9 (44.4%)	3/9 (33.3%)	20/36 (55.6%)	148/205 (72.2%)
Home PC team	124/150 (82.7%)	32/46 (69.6%)	4/10 (40%)	3/7 (42.9%)	14/34 (41.2%)	178/248 (71.8%)[c]

Home nursing	71/86 (82.6%)	6/13 (46.2%)	0/1 (0%)	0/3 (0%)	5/7 (71.4%)	82/110 (74.5%)
**Services with confirmed or suspected COVID-19 cases** (n/N, %)[a]	248/264 (93.9%)	60/83 (72.3%)	9/17 (52.9%)	7/17 (41.2%)	33/58 (56.9%)	358/440 (81.4%)[c]
Missing	13	2	-	2	1	18
**Approximate number of confirmed or suspected COVID-19 cases per service**						
Median (IQR)	25.5 (7, 70)	15 (4.5, 35.5)	3 (2, 70)	8 (2, 20)	6 (2, 11)	16 (5.5, 56)
Total	234	61	9	7	33	345[b]
**Services with confirmed or suspected COVID-19 cases** (n/N, %)						
In-patient PC unit	146/158 (92.4%)	30/43 (69.8%)	3/8 (37.5%)	3/5 (60%)	20/34 (58.8%)	203/249 (81.5%)[c]
Hospital PC team	127/129 (98.4%)	20/26 (76.9%)	5/9 (55.6%)	4/9 (44.4%)	26/36 (72.2%)	182/209 (87.1%)
Home PC team	143/151 (94.7%)	35/46 (76.1%)	5/10 (50%)	3/7 (42.9%)	17/34 (50%)	204/249 (81.9%)[c]
Home nursing	82/87 (94.3%)	7/13 (53.8%)	0/1 (0%)	0/3 (0%)	5/7 (71.4%)	94/111 (84.7%)
**Severity of disease in patients with suspected or confirmed COVID-19** (n/N, %)						
Severely ill or dying due mainly to COVID-19	112/248 (45.2%)	13/60 (21.7%)	1/9 (11.1%)	0/7 (0%)	5/33 (15.2%)	131/358 (36.6%)[b]
Pre-existing illnesses/co-morbidities as well as	192/248 (77.4%)	34/60 (56.7%)	3/9 (33.3%)	5/7 (71.4%)	15/33 (45.5%)	250/358 (69.8%)[c]

COVID-19 who are severely ill or dying						
Patients known to service already who now have COVID-19	129/248 (52%)	21/60 (35%)	3/9 (33.3%)	2/7 (28.6%)	12/33 (36.4%)	168/358 (46.9%)[c]
**Services who reported that family members/close friends of patients had suspected or confirmed COVID-19** (n/N, %)[a]	173/257 (67.3%)	39/81 (48.1%)	1/16 (6.3%)	2/17 (11.8%)	22/57 (38.6%)	237/429 (55.2%)[b]
Missing	20	4	1	2	2	29
**Services with staff with suspected or confirmed COVID-19** (n/N, %)[a]	238/262 (90.8%)	55/83 (66.3%)	4/16 (25%)	7/17 (41.2%)	30/58 (51.7%)	335/437 (76.7%)[c]
Missing	15	2	1	2	1	21
**Type of staff with suspected or confirmed COVID-19** (n/N, %)						
Nurses	224/238 (94.1%)	51/55 (92.7%)	3/4 (75%)	7/7 (100%)	22/30 (73.3%)	308/335 (91.9%)[c]
Physicians	161/238 (67.6%)	32/55 (58.2%)	2/4 (50%)	3/7 (42.9%)	15/30 (50%)	214/335 (63.9%)[c]
Allied health professionals, managed	92/238 (38.7%)	11/55 (20%)	3/4 (75%)	1/7 (14.3%)	7/30 (23.3%)	115/335 (34.3%)[c]
Reception/administrative staff	74/238 (31.1%)	9/55 (16.4%)	0/4 (0%)	2/7 (28.6%)	3/30 (10%)	88/335 (26.3%)[b]
Managers	69/238 (29%)	8/55 (14.5%)	0/4 (0%)	1/7 (14.3%)	0/30 (0%)	78/335 (23.3%)[b]
Others	48/238 (20.2%)	5/55 (9.1%)	1/4 (25%)	0/7 (0%)	4/30 (13.3%)	58/335 (17.3%)[b]
**Services who had volunteers with**						

**suspected or confirmed COVID-19** (n/N, %)[a]						
Yes	33/236 (14%)	3/81 (3.7%)	1/16 (6.3%)	0/17 (0%)	1/56 (1.8%)	38/407 (9.3%)[b]
Missing	41	4	1	2	3	51
**Service changes in response to COVID-19**						
**Services who had changed in response to COVID-19** (n/N, %)[a]	255/263 (97%)	74/82 (90.2%)	14/17 (82.4%)	15/17 (88.2%)	53/57 (93%)	412/437 (94.3%)[c]
Missing	14	3	-	2	2	21
**Services changes in response to COVID-19: level of busyness** (n/N, %)						
A lot more busy	70/255 (27.5%)	27/74 (36.5%)	3/14 (21.4%)	3/15 (20%)	11/53 (20.8%)	114/412 (27.7%)[b]
Slightly more busy	61/255 (23.9%)	21/74 (28.4%)	1/14 (7.1%)	5/15 (33.3%)	9/53 (17%)	97/412 (23.5%)[b]
About the same	49/255 (19.2%)	12/74 (16.2%)	2/14 (14.3%)	4/15 (26.7%)	14/53 (26.4%)	82/412 (19.9%)[c]
Slightly less busy	48/255 (18.8%)	10/74 (13.5%)	4/14 (28.6%)	3/15 (20%)	11/53 (20.8%)	76/412 (18.4%)[b]
Much less busy	27/255 (10.6%)	4/74 (5.4%)	4/14 (28.6%)	0/15 (0%)	8/53 (15.1%)	43/412 (10.4%)[b]
**Services who had lost staff from their service because they have been moved to help the NHS elsewhere** (n/N, %)	85/253 (33.6%)	14/73 (19.2%)	4/13 (30.8%)	4/15 (26.7%)	12/53 (22.6%)	119/408 (29.2%)[b]
**Services who reported that staff offered to help their service from health services elsewhere** (n/N, %)	120/253 (47.4%)	13/73 (17.8%)	0/13 (0%)	3/15 (20%)	9/52 (17.3%)	145/407 (35.6%)[b]

**Services who reported changing how their staff work** (n/N, %)	240/254 (94.5%)	64/73 (87.7%)	12/14 (85.7%)	13/15 (86.7%)	44/53 (83%)	374/410 (91.2%)[c]
**Services who reported changing where their staff work (e.g. home working)** (n/N, %)	217/253 (85.8%)	43/71 (60.6%)	7/14 (50%)	10/15 (66.7%)	41/53 (77.4%)	319/407 (78.4%)[c]
**Services who reported changing how their volunteers engage and where** (n/N, %)	160/223 (71.7%)	46/71 (64.8%)	9/13 (69.2%)	13/15 (86.7%)	39/51 (76.5%)	268/374 (71.7%)[c]
**Use of virtual technologies**						
**Services that reported use of virtual technologies (e.g zoom/teams) with patients and familie**s (n/N, %)						
A lot more	149/252 (59.1%)	25/71 (35.2%)	6/13 (46.2%)	6/15 (40%)	33/53 (62.3%)	219/405 (54.1%)[b]
Slightly more	71/252 (28.2%)	24/71 (33.8%)	2/13 (15.4%)	8/15 (53.3%)	14/53 (26.4%)	120/405 (29.6%)[c]
About the same	29/252 (11.5%)	19/71 (26.8%)	1/13 (7.7%)	0/15 (0%)	6/53 (11.3%)	55/405 (13.6%)[b]
Slightly less	-	-	-	-	-	-
Much less	3/252 (1.2%)	3/71 (4.2%)	4/13 (30.8%)	1/15 (6.7%)	0/53 (0%)	11/405 (2.7%)[b]
**Services that reported use of virtual technologies (e.g zoom/teams) with colleagues** (n/N, %)						

A lot more	236/254 (92.9%)	46/72 (63.9%)	10/14 (71.4%)	8/15 (53.3%)	45/53 (84.9%)	345/409 (84.4%)[b]
Slightly more	14/254 (5.5%)	14/72 (19.4%)	3/14 (21.4%)	5/15 (33.3%)	8/53 (15.1%)	45/409 (11%)[c]
About the same	2/254 (0.8%)	10/72 (13.9%)	0/14 (0%)	2/15 (13.3%)	0/53 (0%)	14/409 (3.4%)[b]
Slightly less	1/254 (0.4%)	2/72 (2.8%)	0/14 (0%)	0/15 (0%)	0/53 (0%)	3/409 (0.7%)[b]
Much less	1/254 (0.4%)	0/72 (0%)	1/14 (7.1%)	0/15 (0%)	0/53 (0%)	2/409 (0.5%)[b]
**Advance care planning (ACP)**						
**Services that reported that they are now involved directly with patients/families in ACP** (n/N, %)						
A lot more	28/253 (11.1%)	6/72 (8.3%)	2/14 (14.3%)	0/15 (0%)	3/53 (5.7%)	39/408 (9.6%)[b]
Slightly more	75/253 (29.6%)	13/72 (18.1%)	1/14 (7.1%)	3/15 (20%)	5/53 (9.4%)	97/408 (23.8%)[b]
About the same	128/253 (50.6%)	45/72 (62.5%)	6/14 (42.9%)	10/15 (66.7%)	43/53 (81.1%)	233/408 (57.1%)[c]
Slightly less	13/253 (5.1%)	6/72 (8.3%)	0/14 (0%)	1/15 (6.7%)	1/53 (1.9%)	21/408 (5.1%)[b]
Much less	9/253 (3.6%)	2/72 (2.8%)	5/14 (35.7%)	1/15 (6.7%)	1/53 (1.9%)	18/408 (4.4%)[b]
**Services that reported that they are now involved advising/supporting others and/or educating about ACP** (n/N, %)						

A lot more	75/253 (29.6%)	11/70 (15.7%)	3/14 (21.4%)	4/15 (26.7%)	8/53 (15.1%)	101/406 (24.9%)[b]
Slightly more	84/253 (33.2%)	19/70 (27.1%)	1/14 (7.1%)	4/15 (26.7%)	11/53 (20.8%)	120/406 (29.6%)[c]
About the same	75/253 (29.6%)	35/70 (50%)	5/14 (35.7%)	6/15 (40%)	31/53 (58.5%)	152/406 (37.4%)[b]
Slightly less	12/253 (4.7%)	3/70 (4.3%)	0/14 (0%)	0/15 (0%)	1/53 (1.9%)	16/406 (3.9%)[b]
Much less	7/253 (2.8%)	2/70 (2.9%)	5/14 (35.7%)	1/15 (6.7%)	2/53 (3.8%)	17/406 (4.2%)[b]
**Bereavement support**						
**Bereavement support** (n/N, %)						
A lot more	32/193 (16.6%)	2/53 (3.8%)	1/13 (7.7%)	0/12 (0%)	2/41 (4.9%)	37/313 (11.8%)[b]
Slightly more	49/193 (25.4%)	14/53 (26.4%)	1/13 (7.7%)	5/12 (41.7%)	9/41 (22%)	78/313 (24.9%)[b]
About the same	76/193 (39.4%)	24/53 (45.3%)	5/13 (38.5%)	2/12 (16.7%)	21/41 (51.2%)	129/313 (41.2%)[c]
Slightly less	23/193 (11.9%)	9/53 (17%)	1/13 (7.7%)	4/12 (33.3%)	6/41 (14.6%)	43/313 (13.7%)[b]
Much less	13/193 (6.7%)	4/53 (7.5%)	5/13 (38.5%)	1/12 (8.3%)	3/41 (7.3%)	26/313 (8.3%)[b]
**Other support**						
**Services that reported encountering patients or families with COVID-19 who are from black and minority ethnic groups** (n/N, %)[a]	93/254 (36.6%)	16/76 (21.1%)	3/16 (18.8%)	5/16 (31.3%)	15/56 (26.8%)	132/419 (31.5%)[b]
Missing	23	9	1	3	3	39
**Changes in specific services**						

**Services that reported changes in in-patient beds in their service** (n/N, %)	117/255 (45.9%)	25/74 (33.8%)	4/14 (28.6%)	4/15 (26.7%)	20/53 (37.7%)	170/412 (41.3%)[b]
**Changes in in-patient beds in your services**						
Number of beds (n/N, %)						
Increased	41/117 (35%)	5/28 (17.9%)	1/4 (25%)	0/4 (0%)	1/21 (4.8%)	48/174 (27.6%)
Stayed about the same	34/117 (29.1%)	10/28 (35.7%)	2/4 (50%)	4/4 (100%)	7/21 (33.3%)	57/174 (32.8%)
Decreased	42/117 (35.9%)	13/28 (46.4%)	1/4 (25%)	0/4 (0%)	13/21 (61.9%)	69/174 (39.7%)
**Services that reported changes in how they provide support for patients in acute hospitals** (n/N, %)	103/255 (40.4%)	22/74 (29.7%)	6/14 (42.9%)	8/15 (53.3%)	30/53 (56.6%)	169/412 (41%)[b]
**Number of patients needing support in acute hospital** (n/N, %)						
Increased	44/101 (43.6%)	8/25 (32%)	2/6 (33.3%)	1/10 (10%)	9/31 (29%)	64/173 (37%)
Stayed about the same	28/101 (27.7%)	4/25 (16%)	2/6 (33.3%)	4/10 (40%)	13/31 (41.9%)	51/173 (29.5%)
Decreased	29/101 (28.7%)	13/25 (52%)	2/6 (33.3%)	5/10 (50%)	9/31 (29%)	58/173 (33.5%)
**In acute hospital, face to face contact with patients/their family members is** (n/N, %)						
A lot more	9/103 (8.7%)	2/25 (8%)	0/6 (0%)	0/10 (0%)	0/32 (0%)	11/176 (6.3%)

Slightly more	6/103 (5.8%)	2/25 (8%)	0/6 (0%)	0/10 (0%)	2/32 (6.3%)	10/176 (5.7%)
About the same	14/103 (13.6%)	2/25 (8%)	1/6 (16.7%)	1/10 (10%)	3/32 (9.4%)	21/176 (11.9%)
Slightly less	33/103 (32%)	5/25 (20%)	1/6 (16.7%)	3/10 (30%)	8/32 (25%)	50/176 (28.4%)
Much less	41/103 (39.8%)	14/25 (56%)	4/6 (66.7%)	6/10 (60%)	19/32 (59.4%)	84/176 (47.7%)
**In acute hospital, face to face contact with staff is** (n/N, %)						
A lot more	29/103 (28.2%)	2/25 (8%)	0/6 (0%)	1/10 (10%)	0/32 (0%)	32/176 (18.2%)
Slightly more	28/103 (27.2%)	2/25 (8%)	0/6 (0%)	0/10 (0%)	1/32 (3.1%)	31/176 (17.6%)
About the same	15/103 (14.6%)	4/25 (16%)	0/6 (0%)	2/10 (20%)	12/32 (37.5%)	33/176 (18.8%)
Slightly less	16/103 (15.5%)	11/25 (44%)	3/6 (50%)	4/10 (40%)	7/32 (21.9%)	41/176 (23.3%)
Much less	15/103 (14.6%)	6/25 (24%)	3/6 (50%)	3/10 (30%)	12/32 (37.5%)	39/176 (22.2%)
**In acute hospital, telephone/remote connection is** (n/N, %)						
A lot more	66/104 (63.5%)	16/25 (64%)	1/6 (16.7%)	7/10 (70%)	17/31 (54.8%)	107/176 (60.8%)
Slightly more	25/104 (24%)	8/25 (32%)	4/6 (66.7%)	3/10 (30%)	11/31 (35.5%)	51/176 (29%)
About the same	11/104 (10.6%)	1/25 (4%)	1/6 (16.7%)	0/10 (0%)	3/31 (9.7%)	16/176 (9.1%)
Slightly less	1/104 (1%)	0/25 (0%)	0/6 (0%)	0/10 (0%)	0/31 (0%)	1/176 (0.6%)

Much less	1/104 (1%)	0/25 (0%)	0/6 (0%)	0/10 (0%)	0/31 (0%)	1/176 (0.6%)
**Services that reported changes in how they provide support for patients in their own homes** (n/N, %)	152/255 (59.6%)	32/74 (43.2%)	8/14 (57.1%)	6/15 (40%)	30/53 (56.6%)	228/412 (55.3%)[b]
**Number of patients needing support in their own homes** (n/N, %)						
Increased	60/152 (39.5%)	15/32 (46.9%)	1/8 (12.5%)	4/6 (66.7%)	13/31 (41.9%)	93/229 (40.6%)
Stayed about the same	72/152 (47.4%)	11/32 (34.4%)	4/8 (50%)	1/6 (16.7%)	15/31 (48.4%)	103/229 (45%)
Decreased	20/152 (13.2%)	6/32 (18.8%)	3/8 (37.5%)	1/6 (16.7%)	3/31 (9.7%)	33/229 (14.4%)
**Face to face contact with patients/their family members in their own homes is** (n/N, %)						
A lot more	4/153 (2.6%)	1/31 (3.2%)	1/8 (12.5%)	0/6 (0%)	3/31 (9.7%)	9/229 (3.9%)
Slightly more	2/153 (1.3%)	4/31 (12.9%)	0/8 (0%)	0/6 (0%)	2/31 (6.5%)	8/229 (3.5%)
About the same	8/153 (5.2%)	5/31 (16.1%)	1/8 (12.5%)	1/6 (16.7%)	1/31 (3.2%)	16/229 (7%)
Slightly less	38/153 (24.8%)	6/31 (19.4%)	2/8 (25%)	1/6 (16.7%)	9/31 (29%)	56/229 (24.5%)
Much less	101/153 (66%)	15/31 (48.4%)	4/8 (50%)	4/6 (66.7%)	16/31 (51.6%)	140/229 (61.1%)
**Face to face contact with staff in patients' homes is** (n/N, %)						

A lot more	2/150 (1.3%)	0/31 (0%)	1/8 (12.5%)	0/6 (0%)	1/29 (3.4%)	4/224 (1.8%)
Slightly more	5/150 (3.3%)	2/31 (6.5%)	0/8 (0%)	0/6 (0%)	1/29 (3.4%)	8/224 (3.6%)
About the same	24/150 (16%)	5/31 (16.1%)	3/8 (37.5%)	1/6 (16.7%)	5/29 (17.2%)	38/224 (17%)
Slightly less	48/150 (32%)	9/31 (29%)	2/8 (25%)	2/6 (33.3%)	10/29 (34.5%)	71/224 (31.7%)
Much less	71/150 (47.3%)	15/31 (48.4%)	2/8 (25%)	3/6 (50%)	12/29 (41.4%)	103/224 (46%)
**For patients in their own homes, telephone/remote connection is** (n/N, %)						
A lot more	125/151 (82.8%)	21/32 (65.6%)	2/8 (25%)	5/6 (83.3%)	22/31 (71%)	175/228 (76.8%)
Slightly more	20/151 (13.2%)	9/32 (28.1%)	4/8 (50%)	0/6 (0%)	6/31 (19.4%)	39/228 (17.1%)
About the same	6/151 (4%)	2/32 (6.3%)	1/8 (12.5%)	1/6 (16.7%)	2/31 (6.5%)	12/228 (5.3%)
Much less	0/151 (0%)	0/32 (0%)	1/8 (12.5%)	0/6 (0%)	1/31 (3.2%)	2/228 (0.9%)
**Services that reported changes in how medicines are given in the community (e.g. who sets up syringe drivers/families administering medicines** (n/N, %)	56/149 (37.6%)	7/31 (22.6%)	1/8 (12.5%)	2/6 (33.3%)	9/28 (32.1%)	75/222 (33.8%)
**Services that reported changes in how they provide support for patients in care homes**	96/255 (37.6%)	23/74 (31.1%)	2/14 (14.3%)	4/15 (26.7%)	28/53 (52.8%)	153/412 (37.1%)[b]

**(including nursing homes**) (n/N, %)						
**Number of patients needing support in care homes (including nursing homes)** (n/N, %)						
Increased	40/93 (43%)	7/23 (30.4%)	0/2 (0%)	2/3 (66.7%)	5/25 (20%)	54/146 (37%)
Stayed about the same	45/93 (48.4%)	7/23 (30.4%)	1/2 (50%)	1/3 (33.3%)	14/25 (56%)	68/146 (46.6%)
Decreased	8/93 (8.6%)	9/23 (39.1%)	1/2 (50%)	0/3 (0%)	6/25 (24%)	24/146 (16.4%)
**Face to face contact with patients/their family members in care homes (including nursing homes) is** (n/N, %)						
A lot more	6/94 (6.4%)	3/21 (14.3%)	0/2 (0%)	0/3 (0%)	0/25 (0%)	9/145 (6.2%)
Slightly more	6/94 (6.4%)	0/21 (0%)	0/2 (0%)	0/3 (0%)	1/25 (4%)	7/145 (4.8%)
About the same	12/94 (12.8%)	0/21 (0%)	1/2 (50%)	0/3 (0%)	0/25 (0%)	13/145 (9%)
Slightly less	18/94 (19.1%)	5/21 (23.8%)	0/2 (0%)	2/3 (66.7%)	4/25 (16%)	29/145 (20%)
Much less	52/94 (55.3%)	13/21 (61.9%)	1/2 (50%)	1/3 (33.3%)	20/25 (80%)	87/145 (60%)
**Face to face contact with staff in care homes (including nursing homes) is** (n/N, %)						
A lot more	8/93 (8.6%)	2/21 (9.5%)	0/2 (0%)	2/3 (66.7%)	0/25 (0%)	12/144 (8.3%)

Slightly more	7/93 (7.5%)	2/21 (9.5%)	0/2 (0%)	0/3 (0%)	1/25 (4%)	10/144 (6.9%)
About the same	7/93 (7.5%)	3/21 (14.3%)	1/2 (50%)	1/3 (33.3%)	2/25 (8%)	14/144 (9.7%)
Slightly less	15/93 (16.1%)	2/21 (9.5%)	1/2 (50%)	0/3 (0%)	4/25 (16%)	22/144 (15.3%)
Much less	56/93 (60.2%)	12/21 (57.1%)	0/2 (0%)	0/3 (0%)	18/25 (72%)	86/144 (59.7%)
**For patients in care homes (including nursing homes), telephone/remote connection is** (n/N, %)						
A lot more	57/93 (61.3%)	14/23 (60.9%)	0/2 (0%)	2/3 (66.7%)	10/25 (40%)	83/146 (56.8%)
Slightly more	20/93 (21.5%)	6/23 (26.1%)	1/2 (50%)	1/3 (33.3%)	7/25 (28%)	35/146 (24%)
About the same	11/93 (11.8%)	2/23 (8.7%)	0/2 (0%)	0/3 (0%)	7/25 (28%)	20/146 (13.7%)
Slightly less	4/93 (4.3%)	1/23 (4.3%)	1/2 (50%)	0/3 (0%)	1/25 (4%)	7/146 (4.8%)
Much less	1/93 (1.1%)	0/23 (0%)	0/2 (0%)	0/3 (0%)	0/25 (0%)	1/146 (0.7%)
**Changes to how you are supporting families/those important to patients**						
**Support for families/ those important to patients compared to before is** (n/N, %)						
A lot more	43/247 (17.4%)	5/66 (7.6%)	1/13 (7.7%)	2/15 (13.3%)	12/47 (25.5%)	63/388 (16.2%)
Slightly more	49/247 (19.8%)	16/66 (24.2%)	3/13 (23.1%)	3/15 (20%)	5/47 (10.6%)	76/388 (19.6%)

About the same	82/247 (33.2%)	24/66 (36.4%)	3/13 (23.1%)	5/15 (33.3%)	18/47 (38.3%)	132/388 (34%)
Slightly less	57/247 (23.1%)	14/66 (21.2%)	2/13 (15.4%)	3/15 (20%)	6/47 (12.8%)	82/388 (21.1%)
Much less	16/247 (6.5%)	7/66 (10.6%)	4/13 (30.8%)	2/15 (13.3%)	6/47 (12.8%)	35/388 (9%)
**Services that reported that they had changed how they contact and work with families/those important to patients** (n/N, %)	217/243 (89.3%)	44/66 (66.7%)	10/13 (76.9%)	11/14 (78.6%)	37/48 (77.1%)	319/384 (83.1%)
**Changes to how you are deploying volunteers**						
**Services that reported changes to how they deploy volunteers** (n/N, %)	155/194 (79.9%)	29/51 (56.9%)	6/10 (60%)	13/15 (86.7%)	33/43 (76.7%)	236/313 (75.4%)
**Deployment of volunteers compared to before is** (n/N, %)						
A lot more	5/157 (3.2%)	3/32 (9.4%)	0/7 (0%)	0/14 (0%)	2/34 (5.9%)	10/244 (4.1%)
Slightly more	4/157 (2.5%)	2/32 (6.3%)	0/7 (0%)	2/14 (14.3%)	0/34 (0%)	8/244 (3.3%)
About the same	7/157 (4.5%)	1/32 (3.1%)	0/7 (0%)	0/14 (0%)	1/34 (2.9%)	9/244 (3.7%)
Slightly less	15/157 (9.6%)	2/32 (6.3%)	3/7 (42.9%)	1/14 (7.1%)	3/28 (8.8%)	24/244 (9.8%)
Much less	126/157 (80.3%)	24/32 (75%)	4/7 (57.1%)	11/14 (78.6%)	28/34 (82.4%)	193/244 (79.1%)
**Shortages**						
**Personal Protective Equipment (PPE) shortages** (n/N, %)[a]	129/258 (50%)	38/75 (50.7%)	9/15 (60%)	6/17 (35.3%)	19/54 (35.2%)	201/419 (48%)

Missing or not applicable[d]	148	47	8	13	40	256
**PPE shortages in the last 7 days** (n/N, %)[e]	50/127 (39.4%)	7/38 (18.4%)	7/9 (77.8%)	5/6 (83.3%)	6/19 (31.6%)	75/199 (37.7%)
Missing or not applicable[d]	227	78	10	14	53	382
**Key medicines shortages** (n/N, %)[a]	63/255 (24.7%)	19/73 (26%)	4/15 (26.7%)	4/17 (23.5%)	11/54 (20.4%)	101/414 (24.4%)
Missing or not applicable[d]	214	66	13	15	48	356
**Key medicines shortages in the last 7 days** (n/N, %)[e]	28/63 (44.4%)	3/18 (16.7%)	4/4 (100%)	4/4 (100%)	6/11 (54.5%)	45/100 (45%)
Missing or not applicable[d]	249	82	13	15	53	412
**Equipment shortages (e.g. syringe drivers)** (n/N, %)[a]	45/256 (17.6%)	3/73 (4.1%)	4/14 (28.6%)	2/17 (11.8%)	2/54 (3.7%)	56/414 (13.5%)
Missing or not applicable[d]	232	82	13	17	57	401
**Equipment shortages in the last 7 days** (n/N, %)[e]	19/42 (45.2%)	1/3 (33.3%)	3/3 (100%)	2/2 (100%)	2/2 (100%)	27/52 (51.9%)
Missing or not applicable[d]	258	84	14	17	57	430
**Staff shortages** (n/N, %)[a]	117/255 (45.9%)	26/75 (34.7%)	4/14 (28.6%)	6/16 (37.5%)	13/54 (24.1%)	166/414 (40.1%)
Missing or not applicable[d]	160	59	13	13	46	291
**Staff shortages in the last 7 days** (n/N, %)[e]	61/114 (53.5%)	9/26 (34.6%)	3/4 (75%)	5/5 (100%)	5/13 (38.5%)	83/162 (51.2%)
Missing or not applicable[d]	216	76	14	14	54	374

59 of the 458 responses completed data but did not hit the final submit button, so lack a date and time stamp. Many were contacted and had no further information. Only 4/458 responders chose not to provide an email address on registration.

Note: UK = United Kingdom, Rest of Europe excludes UK, LIC = Low Income Countries, LMIC = Lower Middle Income Countries, UMIC = Upper Middle Income Countries, HIC = High Income countries, IQR = Interquartile range, NHS = National Health Service, PC = Palliative Care
[a] n of value and valid N denominator are provided. Percentages are of valid values, unless otherwise stated. Number of missing responses for each category are provided.
[b] Includes data from the one missing country in the denominator.
[c] Included data from the one missing country in the numerator and denominator
[d] Not applicable includes those services who did not have shortages
[e] Response for “yes” and “sometimes” both coded as “yes”

Table S 2 Models of care in overall, charity/non-profit and public palliative care settings and numbers of services at only one, only two, only three or all of the settings

Settings and Setting Combinations only at the stated settings	Numbers of services according to management type at specified settings[a]			
	Overall (n=458)	Charity/ Non-Profit (n=192)	Public (n=204)	Other/Private/ Unspecified (n=62)
**Inpatient Palliative Care Unit**	59	31	19	9
Inpatient Palliative Care Unit & Hospital Palliative Care Team	23	6	12	5
Inpatient Palliative Care Unit & Home Palliative Care Team	40	31	5	4
Inpatient Palliative Care Unit & Home Nursing	14	13	0	1
Inpatient Palliative Care Unit & Home Palliative Care Team & Home Nursing	47	42	1	4
Inpatient Palliative Care Unit & Hospital Palliative Care Team & Home Nursing	1	1	0	0
Inpatient Palliative Care Unit & Hospital Palliative Care Team & Home Palliative Care Team	53	12	27	14
Inpatient Palliative Care Unit & Hospital Palliative Care Team & Home Palliative Care Team & Home Nursing	24	18	3	3
**Hospital Palliative Care Team**	85	2	77	6
Hospital Palliative Care Team & Home Palliative Care Team	26	0	21	5
Hospital Palliative Care Team & Home Nursing	0	0	0	0
Hospital Palliative Care Team & Home Palliative Care Team & Home Nursing	5	1	3	1
**Home Palliative Care Team**	48	16	25	7
Home Palliative Care Team & Home Nursing	18	11	5	2

**Home Nursing**	10	6	4	0

[a] Data is missing for 2 Non-Profit, 2 Public and 1 Other/Private/Unspecified service

Table S 3 Overall impact of COVID-19 on palliative care services, main themes and example quotes

**IMPACT OF COVID-19 ON PALLIATIVE CARE SERVICES**
**EMOTIONAL IMPACT**<br><br>**Fatigue**<br><br>*'Emotional fatigue because of a different type of dying & volume, as well as a lack of other type of work'* UK, Other (part of an NHS acute trust)<br><br>'*fatigue - at times - with rapid changes and maintaining a flexible response to change'* UK, Charitable/Non-Profit<br><br>*'Volunteer death, staff family members' severe illness and also one death, and a local GP's death have hit hard. Fatigue has been a feature …tearfulness and also staff not wanting to be away/wanting to give.'* UK, Charitable/Non-Profit<br><br>*'Very intense way of working - no separation of work and home environment- radical changes to practice and assurance of retention of quality and safety for staff and patients has been very demanding…'* Rest of Europe, Charitable/Non-Profit<br><br>**Anxiety and Worry**<br><br>*'…staff have been anxious about their own health and the health of their families…have suffered bereavements and some have been hospitalised themselves, this undoubtedly has an impact on team morale'* UK, Charitable/Non-profit<br><br>*'…some normally resilient and balanced staff have been affected psychologically by health anxieties and not able to function or asking to be redeployed into non patient facing roles.'* UK, Charitable/Non-Profit<br><br>*'…just the feeling of I can be dangerous for patients in palliative care, because most time I am working in acute hospital...'* Rest of Europe, Public<br><br>**Stress & Distress**<br>*'…great moral distress in supporting families who are stressed or bereaved that they cannot or could not be with their loved one through death or visit while the client could still communicate.*' Rest of the World (HIC), Public

‘Emotional burden on staff… ethical dilemmas… tested resilience, in some cases eroded’ UK, Charitable/Non-Profit

‘Working with the on-going uncertainty and adjusting to new ways of working has put an emotional strain and pressure on staff, but it has also provided a strength of purpose and commitment.’ UK, Charitable/Non-Profit

‘Staff are more concerned about how to provide care safely and upset that we are not able to offer the same care we would normally’ UK, Charitable/Non-Profit

‘Increased stress has been evident requiring additional support from clinical psychology’ UK, Charitable/Non-Profit

‘Chronic strain re emotional/social restrictions, constant change and uncertainty’ UK, Charitable/Non-Profit

Guilt

‘For all that I have been less busy than my acute colleagues - and I feel guilty about that …a kind of reverse survivor's guilt…’ UK, Charitable/Non-Profit

‘Staff members becoming ill generates lots of fear and anxiety. Staff feeling guilty if they think they have passed on Covid to patients.’ UK, Public

FRUSTRATION

Changing policy and practice

‘Increased anxiety, and needing to ensure clear communication of changes in policy or practice’ UK, Charitable/Non-Profit

‘…there has been such difficulty coping with the information overload, sifting through what is essential and what is not. The bed capacity tracker has been a very laborious, time consuming and stressful exercise for all involved but at the same time a necessary evil in order to ensure funding.’ UK, Charitable/Non-Profit

'…with the ever changing guidelines, just become familiar and they change again. Changing the service provision and supporting staff through this …everyone is struggling, so it is really difficult to see who to go to for support' UK, Charitable/Non-Profit<br><br>*'…there was a lot of irritation and insecurity, the regional government changed directives nearly every day, because nobody knew what was best to do. We had a lot of work to implement the frequent changing guideline into our hospital and on the same time to teach the staff appropriately.'* Rest of Europe, Public<br><br>**Lack of Sympathy**<br>*'…lack of understanding of acute colleagues of the difficulties facing palliative providers in the community.'* Rest of the World (HIC), Public<br><br>*'…staff on furlough and the animosity this has caused with staff who are still working…'* UK, Charitable/Non-Profit
**FINANCIAL**<br><br>*'Financial distress of hospice resulting in staff taking voluntary pay cuts of up to 30%...'*Rest of the World (UMIC), Charitable/Non-Profit<br><br>*'…fear for oneself and ones patients uncertainty of having a job after this no funding how to sustain the organization as it is a NGO…'* Rest of the World (UMIC), Charitable/Non-Profit

Table S 4 Univariable analysis of potential predictors of PPE shortages

		Odds Ratio	95% CI		p-value	Number of responses in model
**Unit management type**	Public	Ref				415
	Charitable	5.91	3.78	9.24	<0.001	
	Other	1.39	0.69	2.82	0.36	
**Type of patients**	Adults only	Ref				413
	Mixed	1.35	0.74	2.48	0.33	
	Children only	1.15	0.52	2.54	0.74	
**Busyness**	Same	Ref				419
	A lot more busy	0.91	0.52	1.59	0.73	
	Slightly more busy	0.57	0.32	1.02	0.06	
	Slightly less busy	0.75	0.41	1.37	0.34	
	Much less busy	0.32	0.15	0.69	0.003	
**Country**	UK	Ref				419
	Rest of Europe	1.03	0.61	1.72	0.92	
	Rest of the World	0.65	0.40	1.07	0.10	
**Any suspected or confirmed COVID patients**	No	Ref				416
	Yes	1.20	0.73	1.97	0.47	
**Inpatient palliative care unit**	No	Ref				419
	Yes	3.95	2.61	5.96	<0.001	
**Hospital palliative care team**	No	Ref				419
	Yes	0.29	0.20	0.44	<0.001	
**Home palliative care team**	No	Ref				419
	Yes	1.58	1.07	2.33	0.02	
**Home nursing**	No	Ref				419
	Yes	3.53	2.21	5.66	<0.001	

Note Ref = reference category

Table S 5 Univariable analysis of potential predictors of medicines shortages

		Odds Ratio	95% CI		p-value	Number of responses in model
**Unit management type**	Public	Ref				410
	Charitable	0.73	0.45	1.18	0.20	
	Other	1.03	0.48	2.22	0.94	
**Type of patients**	Adults only	Ref				408
	Mixed	0.68	0.31	1.45	0.32	
	Children only	0.53	0.18	1.59	0.26	
**Busyness**	Same	Ref				414
	A lot more busy	1.24	0.66	2.35	0.50	
	Slightly more busy	1.12	0.58	2.17	0.74	
	Slightly less busy	0.81	0.39	1.68	0.57	
	Much less busy	0.57	0.22	1.46	0.24	
**Country**	UK	Ref				414
	Rest of Europe	1.07	0.59	1.94	0.82	
	Rest of the World	0.86	0.48	1.55	0.62	
**Any suspected or confirmed COVID patients**	No	Ref				411
	Yes	1.36	0.73	2.52	0.33	
**Inpatient palliative care unit**	No	Ref				414
	Yes	0.66	0.42	1.04	0.07	
**Hospital palliative care team**	No	Ref				414
	Yes	1.23	0.78	1.93	0.37	
**Home palliative care team**	No	Ref				414
	Yes	1.34	0.85	2.11	0.21	
**Home nursing**	No	Ref				414
	Yes	0.70	0.41	1.21	0.20	

Note: Ref = reference category

Table S 6 Univariable analysis of potential predictors of other equipment shortages

		Odds Ratio	95% CI		p-value	Number of responses in model
**Unit management type**	Public	Ref				410
	Charitable	0.56	0.31	1.04	0.07	
	Other	0.51	0.17	1.54	0.24	
**Type of patients**	Adults only	Ref				408
	Mixed	0.57	0.20	1.67	0.31	
	Children only	0.50	0.11	2.18	0.36	
**Busyness**	Same	Ref				414
	A lot more busy	10.27	3.01	35.07	<0.001	
	Slightly more busy	5.57	1.55	19.96	0.008	
	Slightly less busy	3.41	0.87	13.30	0.08	
	Much less busy	1.40	0.22	8.68	0.72	
**Country**	UK	Ref				414
	Rest of Europe	0.20	0.06	0.67	0.009	
	Rest of the World	0.49	0.22	1.08	0.08	
**Any suspected or confirmed COVID patients**	No	Ref				411
	Yes	1.88	0.77	4.58	0.16	
**Inpatient palliative care unit**	No	Ref				414
	Yes	0.38	0.21	0.69	0.001	
**Hospital palliative care team**	No	Ref				414
	Yes	1.20	0.68	2.11	0.52	
**Home palliative care team**	No	Ref				414
	Yes	0.88	0.50	1.55	0.66	
**Home nursing**	No	Ref				414
	Yes	0.77	0.39	1.51	0.44	

Note: Ref = reference category

Table S 7 Univariable analysis of potential predictors of staff shortages

		Odds Ratio	95% CI		p-value	Number of responses in model
**Unit management type**	Public	Ref				410
	Charitable	1.33	0.88	2.01	0.19	
	Other	0.96	0.47	1.97	0.91	
**Type of patients**	Adults only	Ref				408
	Mixed	0.46	0.23	0.92	0.03	
	Children only	0.24	0.08	0.70	0.009	
**Busyness**	Same	Ref				414
	A lot more busy	3.18	1.76	5.74	<0.001	
	Slightly more busy	1.60	0.86	2.96	0.14	
	Slightly less busy	1.52	0.79	2.91	0.21	
	Much less busy	0.92	0.41	2.06	0.85	
**Country**	UK	Ref				414
	Rest of Europe	0.63	0.37	1.07	0.09	
	Rest of the World	0.44	0.26	0.76	0.003	
**Any suspected or confirmed COVID patients**	No	Ref				411
	Yes	2.54	1.41	4.54	0.02	
**Inpatient palliative care unit**	No	Ref				414
	Yes	1.34	0.90	2.00	0.15	
**Hospital palliative care team**	No	Ref				414
	Yes	0.96	0.65	1.42	0.84	
**Home palliative care team**	No	Ref				414
	Yes	1.08	0.73	1.61	0.69	
**Home nursing**	No	Ref				414
	Yes	1.30	0.83	2.03	0.24	

Note: Ref = Reference category

Appendix II

CovPall Questionnaire ©

COVPALL COLLABORATION SURVEY

Improving palliative care for people with COVID-19 by sharing learning

Thank you for agreeing to complete this survey. We are trying to find out about how palliative care and hospice services are changing as a result of the COVID-19 pandemic. This is important as the disease is new and hospices/palliative care services are changing how they work and there is an opportunity to learn from each other.

We realise you are very busy right now, and so we have tried to balance collecting the information that patients, policy makers and services think is most helpful, with keeping the questionnaire as short as we can.

The questionnaire has 7 sections, and should take no longer than 30 minutes to complete, although it may depend on how much additional/open comments you wish to share. We will consider everything that you say. Your reply will help us. Although the grouped results will be shared, we will not name your unit unless you ask us to.

You can pause the questionnaire by clicking the "Save & Return Later" button at the bottom of each page. You will be given a code to enable you to continue later. (There should be a "returning" option at the top right of this page.) If you wish to correct errors after you have clicked "Submit" please email palliativecare@kcl.ac.uk with CovPall in the subject line.

If you would like help in completing the survey, or would prefer someone to read the questions to you while you give the answers over the telephone / zoom call, please send your contact details to palliativecare@kcl.ac.uk with CovPall in the subject line.

If you have any concerns about this questionnaire or this study please email ea_irenehigginson@kcl.ac.uk. CovPall is led by Professor Irene Higginson of the Cicely Saunders Institute, with a multiprofessional team of partners from different organisations and backgrounds. Patient representatives have contributed to this questionnaire and our plans. For more information see https://www.kcl.ac.uk/cicelysaunders (specific page later).

This study has been granted ethical approval by the PNM Research Ethics Subcommittee of King's College London, code LRS-19/20-18541.

Please tick all responses that apply.

1. ABOUT YOU AND YOUR SERVICE

1.1 Contact email of the person completing the survey ________________________________
(We need this information so we can help you get back into the survey, or help you to complete it, if you have difficulty)

1.2 Name of the person completing the survey ________________________________

1.3 Date ________________________________
(DD-MM-YYYY)

1.4 Country

◯ England
◯ Scotland
◯ Wales
◯ N Ireland
◯ Australia
◯ Belgium
◯ Canada
◯ Germany
◯ Ireland
◯ Italy
◯ Poland
◯ New Zealand
◯ Other (a box will open)
(A regions option may appear)

1.4a Country (please specify) ________________________________

1.4b English Regions

◯ North East
◯ North West
◯ Yorkshire and The Humber
◯ East Midlands
◯ West Midlands
◯ East
◯ London
◯ South East
◯ South West

1.4c Welsh region

◯ N Wales
◯ W Wales
◯ SE Wales

1.4d Scottish region

◯ Fife, Lothian, Borders, Dumfries & Galloway
◯ Greater Glasgow & Clyde, Ayrshire & Arran, Lanarkshire, Forth Valley
◯ Tayside, Grampian, Western Isles, Highland, Orkney and Shetland

1.4e Region ________________________________

1.5 Your role

◯ Medical director / lead medical clinician
◯ Nurse director / lead nurse clinician
◯ Other (a box will open below)

1.5a Please specify your role ________________________________

---

2. ABOUT THE SERVICES YOU USUALLY OFFERED BEFORE THE COVID-19 PANDEMIC.

Please tick all answers that apply unless indicted

2.1 Types of patients cared for

☐ Adults ☐ Children

2.2 In what settings did you provide palliative care services

(additional questions will open for each choice)

☐ In-patient hospice / palliative care unit
☐ Hospital palliative care advisory team
☐ Specialist palliative home care service (supporting / consulting about care for patients at home and/or in the community)
☐ Providing hands on nursing care at home / in the community (e.g. hospice@home, pall@home)
(Tick all that apply)

Questions for in-patient hospice / palliative care unit

2.2a Number of beds ______________________
(Must be a number)

2.2b Approximate number of new patients seen annually ______________________
(Must be a number)

2.2c Normal hours of admitting patients ______________________
(E.g. 9:30-17:00)

Questions for hospital palliative care advisory team

2.2d Approximate number of new patients seen annually ______________________
(Must be a number)

2.2e Did you support
- ☐ Acute hospitals
- ☐ Community hospitals

2.2f Normal hours of accepting new referrals ______________________
(E.g. 9:30-17:00)

2.2g Did you offer 24/7 support for your patients ☐ Yes

Questions for specialist palliative home care service

2.2h Approximate number of new patients seen annually ______________________
(Must be a number)

2.2i Did you support patients in care homes ○ Yes ○ No

2.2j Normal hours of accepting new referrals ______________________
(E.g. 9:30-17:00)

2.2k Did you offer 24/7 support for your patients ☐ Yes

Questions for providing hands on nursing care at home / in the community

2.2l Approximate number of new patients seen annually ______________________
(Must be a number)

2.2m Normal hours of accepting new referrals ______________________
(E.g. 9:30-17:00)

2.2n Did you offer 24/7 support for your patients ☐ Yes

Additional information about services offered before the COVID-19 pandemic

2.3 Normal hours of accepting new referrals ________________

2.4 Did you offer bereavement services ◯ Yes ◯ No

(if yes - additional questions will open)

2.4a Bereavement services / support usually provided and to whom ________________

2.4b Did you offer bereavement services only to families / friends of patients who had been cared for by your service ◯ Yes ◯ No

2.4c Whom did you offer support to ________________

2.4d Did you use a risk assessment tool to help you decide how to target bereavement services ◯ Yes ◯ No

2.5 Other services provided, e.g. Day care, rehabilitation, lymphoedema, outpatient clinics - please detail ________________

2.6 If you had volunteer roles available within your service, what were they

Tick all that apply

- ☐ Direct patient / family facing support (e.g. befriending, home visits, in-patient unit care, family support groups / visiting etc.)
- ☐ Indirect patient / family facing support (e.g. reception functions, refreshments, driving / transport etc.)
- ☐ Back office functions (e.g. finance support, maintenance, gardening etc.)
- ☐ Fundraising functions (e.g. shop volunteers, lottery etc.)
- ☐ Others (a box will open below)

2.6a Please specify the other volunteer roles ________________

2.7 Did you use remote consultations to help support patients in your care or for education before the COVID-19 Pandemic

Tick all that apply (an example box will appear)

- ☐ Telephone support for education
- ☐ Telephone support for clinical care
- ☐ Telehealth / video support / e-learning for education
- ☐ Telehealth / video support / e-learning for clinical care

2.7a Please give a brief example of the use of remote consultations ________________

2.8 Is your service managed as a unit that is

- ◯ Charitable / non-profit
- ◯ Public
- ◯ Private
- ◯ Other (a box will open below)

2.8a Please explain your unit type ________________

2.8b what percentage of your funding was usually from the NHS ________________
(Must be a number (0-100))

2.9 How well would you say your service was integrated with other NHS primary or secondary care services in your area (e.g. with hospitals, primary care etc.)

Please rank the level of integration: from 0 (no integration at all, no connections) to 10 (very well integrated / close working and planning)

0 5 10

(Place a mark on the scale above)

2.10 Any comments on integration ______________________

2.11 Is there anything else you want to tell us about how your service operates that you think is important for us to know ______________________

3. EXPERIENCE WITH SUSPECTED OR CONFIRMED CASES OF COVID-19. Please tick all that apply

Please tell us about people you have encountered with suspected or confirmed COVID-19

3.1 Have you had any patients with confirmed (by test) cases of COVID-19

(additional questions will open if you tick yes)

○ Yes ○ No

3.1a Approximately how many (confirmed cases) ______________________
(By the date of completing this survey)

3.1b Which services were they in

Tick all that apply

- ☐ In-patient hospice / palliative care unit
- ☐ Home palliative care service
- ☐ Acute hospital
- ☐ Care home
- ☐ Other (a box will open below)

(Tick all that apply)

3.1c Please specify the other services ______________________

3.2 Have you had any patients with suspected (untested but with clinical diagnosis/symptoms) of COVID-19

(additional questions will open if you tick yes)

○ Yes ○ No

3.2a Approximately how many (suspected cases) (by the date of completing this survey) ______________________
(Must be a number)

3.2b Which services were they in

- ☐ In-patient hospice / palliative care unit
- ☐ Home palliative care service
- ☐ Acute hospital
- ☐ Care home
- ☐ Other (a box will open below)

(Tick all that apply)

3.2c Please specify the other services ______________________

3.2d Of the patients you have seen with suspected or confirmed COVID-19 would you say that they were

- ☐ Patients who are severely ill or dying due mainly to COVID-19
- ☐ Patients with pre-existing illnesses / co-morbidities as well as COVID-19 who are severely ill or dying
- ☐ Patients known to your service already who now have COVID-19
- ☐

(Tick all that apply)

3.3 Have you had any family members / close friends of your patients who had suspected or confirmed COVID-19

◯ Yes ◯ No

3.4 Have you had staff with suspected or confirmed COVID-19

(if yes - additional questions will open)

◯ Yes ◯ No

3.4a Were the staff

- ☐ Nurses
- ☐ Physicians
- ☐ Allied health professionals, managed
- ☐ Reception / administrative staff
- ☐ Managers
- ☐ Others (a box will open below)

(Tick all that apply)

3.4b Please specify the other staff

____

3.4c What impact has this had on your service

____

3.5 Have you had volunteers with suspected or confirmed COVID-19

(if yes - an additional question will open)

◯ Yes ◯ No

3.5a What impact has this had on your service

____

4. HOW HAVE YOUR SERVICES CHANGED IN RESPONSE TO COVID-19

4.1 Have your services changed

◯ Yes ◯ No

4.2 Would you say overall you are more busy or less busy that before the COVID-19 Pandemic

- ◯ A lot more busy
- ◯ Slightly more busy
- ◯ About the same
- ◯ Slightly less busy
- ◯ Much less busy

(Tick the answer that best applies)

4.3 Why is this

____

4.4 Have you lost staff from your service who have been moved to help the NHS elsewhere

(if yes - a box for details will open)

◯ Yes ◯ No

4.4a Please give details (lost staff)

____

4.5 Have you had staff offered to help your service from health services elsewhere — ◯ Yes ◯ No

(if yes - a box for details will open)

4.5a Please give details (offered staff) __________________________________

4.6 Have you changed how your staff work — ◯ Yes ◯ No

(if yes - a box for details will open)

4.6a Please give details (how work) __________________________________

4.7 Have you changed where your staff work (e.g. home working) — ◯ Yes ◯ No

(if yes - a box for details will open)

4.7a Please give details (where work) __________________________________

4.8 Have you changed how your volunteers engage and where — ◯ Yes ◯ No

(if yes - a box for details will open)

4.8a Please give details (changed volunteers) __________________________________

Use of virtual technologies

4.9 Would you say that you are using virtual technologies (e.g. zoom / teams etc.) with patients and families

◯ A lot more
◯ Slightly more
◯ About the same
◯ Slightly less
◯ Much less
(Tick the answer that best applies)

4.10 Would you say that you are using virtual technologies (e.g. zoom / teams etc.) with colleagues

◯ A lot more
◯ Slightly more
◯ About the same
◯ Slightly less
◯ Much less
(Tick the answer that best applies)

4.11 What have been the difficulties of using virtual technologies __________________________________

4.12 What has worked well when using virtual technologies __________________________________

Advance care planning

4.13 Would you say you are now involved directly with patients / families in advance care planning

◯ A lot more
◯ Slightly more
◯ About the same
◯ Slightly less
◯ Much less
(Tick the answer that best applies)

4.14 Would you say you are now involved advising / supporting others and / or educating about advance care planning

- ◯ A lot more
- ◯ Slightly more
- ◯ About the same
- ◯ Slightly less
- ◯ Much less

(Tick the answer that best applies)

4.15 In what ways (if any) have you changed how you are supporting advance care planning

____

4.16 What would you say are the main challenges for advance care planning during the COVID-19 pandemic

____

Bereavement support

4.17 Would you say that you provide more or less bereavement support than before

- ◯ A lot more
- ◯ Slightly more
- ◯ About the same
- ◯ Slightly less
- ◯ Much less

(Tick the answer that best applies)

4.18 What would you say are the main challenges for bereavement support during the COVID-19 pandemic

____

Other support

4.19 How are you supporting patients with COVID-19 who are from more disadvantaged sociodemographic communities (e.g. areas with greater poverty, poor housing, homelessness)

____

4.20 Have you encountered patients or families with COVID-19 who are from black and minority ethnic groups

◯ Yes ◯ No

(if yes - a box for details of differences will open)

4.20a Are there any differences in how you are supporting or reaching them

____

4.21 Are there any groups (e.g. with different religions, cultures) where you have found supporting the individual needs of people affected by COVID-19 is particularly challenging

____

Effects on patients who do not have COVID-19

4.22 How has COVID-19 changed how you are supporting the types of patients (e.g. with symptoms and progressive illness) that you would usually support __________________________________

4.23 How has COVID-19 changed how you are supporting the families / those important to patients that you would usually support __________________________________

5. CHANGES IN SPECIFIC SERVICES, E.G. IN-PATIENT, HOSPICE, VOLUNTEERS

The next questions ask about some specific changes that might have occurred, please answer only the sections that apply to your services

5.1 Have there been changes in these areas

(additional questions will open for each choice)

- ☐ In-patient beds in your own service
- ☐ How you provide support for patients in acute hospitals
- ☐ How you provide support for patients in their own homes
- ☐ How you provide support for patients in care homes (including nursing homes)

(Tick all that apply)

Changes in in-patient beds in your service

5.1a What changes were there in how you used your beds (if any) __________________________

5.1b Number of beds

- ◯ Increased
- ◯ Stayed about the same
- ◯ Decreased

5.1c Any changes to admission criteria (if so what was the change) __________________________

5.1d Any changes to out of hours admissions (e.g. evenings / weekends - if so what was the change) __________________________

Changes in how you provide support for patients in acute hospitals

5.1e Numbers of patients needing support

- ◯ Increased
- ◯ Stayed about the same
- ◯ Decreased

5.1f Would you say your face to face contact with patients / their family members is in general

- ◯ A lot more
- ◯ Slightly more
- ◯ About the same
- ◯ Slightly less
- ◯ Much less

5.1g Would you say your face to face contact with staff is

◯ A lot more
◯ Slightly more
◯ About the same
◯ Slightly less
◯ Much less

5.1h Would you say your telephone / remote connection advice / support is

◯ A lot more
◯ Slightly more
◯ About the same
◯ Slightly less
◯ Much less

5.1i Have you changed how your team is organized (e.g. supporting patients with and without COVID-19)

____

5.1j Have you changed your working hours (and in what way)

____

5.1k Have you changed your working practices (and in what way)

____

Changes in how you provide support for patients in their own homes

5.1l Numbers of patients needing support

◯ Increased
◯ Stayed about the same
◯ Decreased

5.1m Would you say your face to face contact with patients / their family members is

◯ A lot more
◯ Slightly more
◯ About the same
◯ Slightly less
◯ Much less

5.1n Would you say your face to face contact with staff is

◯ A lot more
◯ Slightly more
◯ About the same
◯ Slightly less
◯ Much less

5.1o Would you say your telephone / remote connection advice / support is

◯ A lot more
◯ Slightly more
◯ About the same
◯ Slightly less
◯ Much less

5.1p Have you changed how your team is organized (e.g. supporting patients with and without COVID-19)

____

5.1q Have you changed your working hours (and in what way)

____

5.1r Have you changed your working practices (and in what way)

____

5.1s Have you changed how medicines are given in the community (e.g. who sets up syringe drivers / families administering medicines)

(if yes - a box for details will open)

◯ Yes ◯ No

5.1t Please give details (changed how medicines are given in the community)

____

Changes in how you provide support for patients in care homes (including nursing homes)

5.1u Numbers of patients needing support

- ◯ Increased
- ◯ Stayed about the same
- ◯ Decreased

5.1v Would you say your face to face contact with patients / their family members is

- ◯ A lot more
- ◯ Slightly more
- ◯ About the same
- ◯ Slightly less
- ◯ Much less

5.1w Would you say your face to face contact with staff is

- ◯ A lot more
- ◯ Slightly more
- ◯ About the same
- ◯ Slightly less
- ◯ Much less

5.1x Would you say your telephone / remote connection advice / support is

- ◯ A lot more
- ◯ Slightly more
- ◯ About the same
- ◯ Slightly less
- ◯ Much less

5.1y Have you changed how your team is organized (e.g. supporting patients with and without COVID-19) __________________________________

5.1z Have you changed your working hours (and in what way) __________________________________

5.1α Have you changed your working practices (and in what way) __________________________________

Changes to how you are supporting families / those important to patients

5.2 How would you say you are supporting families / those important to patients compared to before

- ◯ A lot more
- ◯ Slightly more
- ◯ About the same
- ◯ Slightly less
- ◯ Much less

5.3 Have you changed how you contact and work with families / those important to patients

◯ Yes ◯ No
(If yes, a box for details will open)

5.3a Please give details (changed how contact and work with families)

____

Changes to how you are deploying volunteers

5.4 How would you say you are deploying volunteers compared to before

- ◯ A lot more
- ◯ Slightly more
- ◯ About the same
- ◯ Slightly less
- ◯ Much less

5.5 Have you changed how you deploy volunteers ◯ Yes ◯ No

(if yes - a box for details will open)

5.5a Please give details (changed how contact and work with families) __________________________________

6. CHALLENGES AND INNOVATIONS IN RESPONSE TO COVID-19

We want to know more about the challenges you have faced, their impacts on your services and care, how you have responded to them and what you found to be your successful innovations

Please tick the challenges that you have faced in your service during COVID-19 Pandemic during the past 1 month

6.1 Have you had problems accessing personal protective equipment ◯ Yes ◯ No

(if yes - additional questions will open)

6.1a Please specify what you had a shortage of __________________________________

6.1b What did you do about this __________________________________

6.1c Has this been a problem in the last 7 days
◯ Yes
◯ Sometimes
◯ No

6.2 Have you had a shortage of key medicines ◯ Yes ◯ No

(if yes - additional questions will open)

6.2a Please specify what you had a shortage of __________________________________

6.2b What did you do about this __________________________________

6.2c Has this been a problem in the last 7 days
◯ Yes
◯ Sometimes
◯ No

6.3 Have you had a shortage of other equipment (e.g. syringe drivers) ◯ Yes ◯ No

(if yes - additional questions will open)

6.3a Please specify what you had a shortage of __________________________________

6.3b What did you do about this __________________________________

6.3c Has this been a problem in the last 7 days
- ◯ Yes
- ◯ Sometimes
- ◯ No

6.4 Have you had a shortage of staff ◯ Yes ◯ No

(if yes - additional questions will open)

6.4a Please specify what you had a shortage of

____

6.4b What did you do about this

____

6.4c Has this been a problem in the last 7 days
- ◯ Yes
- ◯ Sometimes
- ◯ No

6.5 Have there been other effects on yourself and/or on staff that you think we should know about

____

6.6 Please tell us about any other challenges and whether or how you overcame them

____

6.7 What do you foresee will be the biggest challenges for COVID-19 in your service over the next 1-2 months

____

6.8 What would help you most to overcome these

____

Now please tell us about your innovations. We are keen to learn what has worked best for you

6.9 Please tell us about the change in practice or innovation that you think has been most successful to your working

____

6.10 Why is this

____

6.11 What would you say were the most important things that made this possible

____

6.12 Please list any other important changes / innovations you have made

____

7. SYMPTOM MANAGEMENT

We are interested to learn more about how you are managing symptoms and psychological / emotional problems in our patients and the trajectories of care

How long are patients with COVID-19 under your palliative care service

7.1 What was the shortest time in hours

____
(Give number of hours)

7.2 What was the longest time in days

____
(Give number of days)

How are you managing symptoms

7.3 Please indicate which of these settings your management refers to (choose only one about which you have most experience)

○ Inpatient hospital ward
○ Inpatient hospital intensive care
○ Community hospital
○ Inpatient hospice / palliative care ward
○ Community

The next questions ask about the treatments you are using

7.4 Do you have protocols for symptom management for COVID-19 patients

○ Yes
○ No
○ Unsure
(Yes will ask for details)

7.4a What sources of information did you use

☐ Locally developed guidance
☐ NICE
☐ NHS
☐ Other (a box will open below)

7.4b Please specify the other protocols

____

You can also email us your usual recommendations of any guidance you provide by email to: palliativecare@kcl.ac.uk , marking the email CovPall in the subject line. We are still interested to know how well you find these treatments are working

Breathlessness

7.5 Which medicines and therapies do you usually prescribe

____

7.6 How effective do you find these e.g. time to give relief and how well it works

____

Agitation

7.7 Which medicines and therapies do you usually prescribe

____

7.8 How effective do you find these e.g. time to give relief and how well it works

____

Fever / Shivering

7.9 Which medicines and therapies do you usually prescribe

____

7.10 How effective do you find these e.g. time to give relief and how well it works

____

Cough

7.11 Which medicines and therapies do you usually prescribe

____

7.12 How effective do you find these e.g. time to give relief and how well it works

____

Pain

7.13 Which medicines and therapies do you usually prescribe

____

7.14 How effective do you find these e.g. time to give relief and how well it works

____

Other symptoms

7.15 Please provide details of the treatments you are using for any other symptoms you are seeing commonly in COVID-19, especially if this differs from usual palliative care practice

____

Additional comments

7.16 Please provide any additional comments you would like us to be aware of ______________________

7.17 Please tell us if you would like us to help you by providing anything else, or any key questions that you think are important to answer ______________________

Finally

7.18 Please indicate if you would like / are willing to be contacted regarding any of the following
(Tick all that apply)

☐ To receive copy of the early reports and our newsletters as the findings emerge
☐ For us to check any information with you
☐ To be acknowledged as responding to this questionnaire (listed along with other services) in the reports and any publications
☐ To participate in a subsequent survey similar to this one in 6-8 weeks time when you may have made more changes or had more experiences
☐ To collect pseudoanonymized data about a small series (around 10) of patients with COVID-19 in your service. This would involve collecting information on symptom severity, on first assessment in palliative care, in around 2 subsequent time points and at discharge or death, to understand more about the symptoms patients experience and their the effective treatments. This would not be a clinical trial, simply recording your practice and views

7.19 If you wish us to use a different Name or Email for the above (instead of the ones already given) please specify here ______________________

7.20 How you would like your service acknowledged in any reports, if applicable ______________________

You will be free to opt out of receiving the updates at any time, your details will not be passed onto other organisations or used for anything other than with your explicit consent above. Your individual responses will remain confidential, they will be analysed pseudonymously by the research team, with your service identified only by a code number unless you explicitly ask us to do otherwise.

Thank you for your help at this difficult time

Appendix III: Acknowledgement of services

We would like to thank all the palliative care services and leads across the world for responding to the CovPall online survey.

The following persons and services indicated they were happy to be acknowledged for responding to the CovPall online survey:

Alexander Devine Children’s Hospice Service
Alison Wardrop District Nursing Sister, Hywel Dda University Health Board
Ashgate Hospicecare
Bangalore Baptist Hospital Palliative Care Service
BCUHB West HSPCT
Beatson West of Scotland Cancer Centre HSPCT
Beaumond House Community Hospice
Betsi Cadwaladr University Health Board SPCT
Birmingham St Mary’s Hospice
Bluebell Wood Children's Hospice
Bolton NHS Foundation Trust Specialist Palliative Care Team
Bury Community Specialist Palliative Care Team
Cambridge University Hospital NHS Foundation Trust Palliative Care Service
Carmarthenshire Specialist Palliative Care Service, Hywel Dda University Health Board
Center for Palliative Care, University Hospital Cologne, Germany
Cesta domů, home hospice
Chelsea and Westminster Hospital
CHFT Hospital SPCT
Children’s hospices across Scotland (CHAS)
City Hospice Cardiff
Claire House Children's Hospice
Clinique de Médecine Palliative, CHU de Lille, France
CNWL UCLH
Community based clinical nurse specialists
Community Specialist Palliative Care Team, St Ann's Hospice, Salford
Consultant Dr Paul Coulter, CNS Elizabeth Anderson and CNS Kellyann O'Neill, Palliative Care Team, Inverclyde Royal Hospital
Cornwall Hospice Care
Croydon Health Services Macmillan Specialist Palliative Care Team

Cwm Taf Morgannwg SPCT North
Department of Palliative Care, Homerton University Hospital NHS Foundation Trust
Department of Palliative care, Sheffield Teaching Hospitals NHS Foundation Trust
Department of Palliative Medicine, LMU Munich, Germany
Department of Palliative Medicine, Southern Sector, South East Sydney Local Health District, New South Wales, Australia
Deutsche PalliativStiftung
Dorothy House Hospice
Dove House Hospice, Hull
Dr Charles Daniels, Medical Director, St Luke's Hospice Harrow and Consultant in Palliative Medicine, LNWHUT.
Dr Jonathan Downie, Consultant in Paediatric Palliative Medicine, Supportive and Palliative Care Team, Royal Hospital for Children, Glasgow
East Anglia's Children's Hospices (EACH)
East Cheshire Hospice
East Sussex Healthcare NHS Trust Supportive and Palliative Care Team
Eastern Health
Ed Dubland MD CCFP pc
Ellenor Adult Services
Ellenor Children's Services
Ellenor Hospice, Coldharbour Road, Gravesend, Kent
Ellenor Inpatient Ward
Ellenor Wellbeing Service
Elvis J Miti for Community Palliative Care, UZIMA Project Ndanda, Mtwara, Tanzania.
ESNEFT specialist palliative care team
Family Support Team, Children's Hospices Across Scotland
Farleigh Hospice
Felicia Kontopidis, Journey Home Hospice
Fondazione Antea, Rome, Italy
Fondazione FARO, Turin, ITALY
Forest Holme Hospice, Poole Hospital NHS Foundation Trust
Forget Me Not Children's Hospice

Garden House Hospice Care
GHNHSFT Specialist Palliative Care Community team
Glasgow Royal Infirmary Hospital Specialist Palliative Care Team
Greenwich & Bexley Community Hospice
GSTT Community Palliative Care Team
Hambleton & Richmondshire SPCT
Hampshire Hospitals NHS Foundation Trust
Harrogate and District NHS Foundation Trust Palliative Care Team
Heart of Kent Hospice
Helen & Douglas House
Hospice De Wingerd (Charim Care Group)
Hospice Isle of Man
Hospice of St. Francis Berkhamsted, Herts
Hospice of The Good Shepherd, Chester
Hospice Waikato
Hospiscare Devon
Hospital Divina Providencia, El Salvador
Hospital Palliative Care Team, Kingston Hospital NHS Foundation Trust
Hospital Palliative Care Team, University Hospitals of North Midlands
Hospital Palliative Care team, University Hospital Southampton
Hospital Selayang Palliative Care Unit
Hospital Specialist Palliative care team
Hospital Specialist Palliative Care Team, University Hospitals of Leicester NHS Trust
Hull University Teaching Hospitals NHS Trust
Instituto de Investigaciones Médicas Alfredo Lanari, Universidad de Buenos Aires
Isabel Hospice
J Yeomans, St Richards Hospice (Worcester)
James Cook University Hospital Acute SPCT
Thitima Phosri
John Taylor Hospice
Helen Hubert, Palliative care and End of Life Care Pharmacist, St Richard’s Palliative Care Team
Katharine House Hospice Banbury

Kauniala Hospital
Kenelm F McCormick M.D., Akron, Ohio
Kilbryde Hospice, South Lanarkshire
Kim Jones, Deputy Head of Clinical Services at Hospice of the Valleys
King's College Hospital NHS Foundation Trust
Klinik Susenberg, Zürich
Knysna Sedgefield Hospice
Lanarkshire SPC service
Laois Offaly Palliative Care Service Cloneygowan Co Offaly
Leeds Teaching Hospitals NHS Trust Palliative Care Team
Limewood Dementia Service, Stafford
London Northwest Healthcare University Trust
LOROS Hospice, Leicester
Lynn Bushor, Department of Veteran's Affairs
Macmillan Unit / Royal Bournemouth & Christchurch
Manchester Foundation Trust - MRI
Manchester Foundation Trust WTWA
Maricruz Macias Montero, Hospital General de Segovia, Seccion de Geriatria.
Marie Curie Community Nursing Service London
Marie Curie Hospice, Cardiff and the Vale
Marie Curie Hospice West Midlands
Marie Curie Hospice, Hampstead
Marie Curie Services in Lothian
Martlets Hospice
Martlets Hospice, Brighton
Maura Farrell Miller, Director, Hospice and Palliative Care Program, Department of Veterans Affairs Medical Center, Florida
Mellannorrlands Hospice Sundsvall Sweden
Michael Sobell Hospice and Harlington Hospice
Mid Cheshire Hospital NHS Trust Specialist Palliative Care Team
Midhurst Macmillan Service (Sussex Community NHS Foundation Trust)
Municipal Hospital Dr Cornel Igna, Palliative Care Department from Campia Turzii

Myton Hospice
Newham University Hospital Specialist Palliative Care Team - Barts Health NHS Trust
NHS Ayrshire & Arran Supportive Care Team
NHS Fife Specialist Palliative Care Service
NHS Grampian Specialist Palliative Care Team
NHS Specialist Palliative Care Unit
Nightingale House Hospice, North Wales
Ninewells Hospital Palliative Care Team
North London Hospice
Northern Ireland Hospice Adult Service
Northumbria Healthcare NHS Foundation Trust
Nottingham University Hospitals NHS Trust
Ofra Fried, Palliative Care Specialist, Townsville University Hospital, Queensland
Overgate Hospice
Palliativa Care Unit. west health Area. Valladolid. Castilla y León .Spain
Palliative and End of Life care Team, Newcastle upon Tyne Hospitals NHS Foundation Trust
Palliative Care Department, Bangalore Baptist Hospital
Palliative Care Service, Chaim Sheba Medical Center, Ramat-Gan, Israel
Palliative Care Service, Gloucestershire Hospitals NHS Foundation Trust
Palliative Care Team-Bassett Medical Center, Cooperstown NY
Palliative Care Team, Centre for Pain Management and Palliative Care, Haukeland University Hospital, Bergen, Norway
Palliative Care Unit, Bolzano, Italy
Palliative Care Unit, Bassini Hospital, Cinisello Balsamo, Milan, Italy
Palliative Care, Kantonsspital Olten, Switzerland
Palliative Medicine, UHDB NHS Trust
Palliaviva
Peace Hospice Care
Pembrokeshire Specialist Palliative Care service, Hywel Dda University Health Board
Phyllis Tuckwell Hospice Care
Pilgrims Hospices in East Kent
Pippa Hawley, Medical Director, Pain and Symptom Management Palliative Care, BC Cancer.

Portsmouth Hospital NHS Trust Palliative Care Service
Princess Alice Hospice
Prof Olaitan A Soyannwo
Queen Elizabeth University Hospital Glasgow Specialist Palliative Care Team, and Prince and Princess of Wales Hospice Glasgow
Queenscourt Supportive & Specialist Palliative Care Service
Rotherham NHS Foundation Trust Palliative Care Team
Rowans Hospice
Rowcroft Hospice
Royal Berkshire Foundation Trust Hospital Palliative Care Team
Royal Trinity Hospice
Sabar Health Home Hospital
Saint Francis Hospice
Salford Royal Hospital Specialist Palliative Care Team
Salisbury Specialist Palliative Care service
SAPV des OSP Karlsruhe
SCCU SEVILLA SUR, SAS. SPAIN.
Sengkang General Hospital, Singapore
Shooting Star Children's Hospice
Sobell House, Oxford University Hospitals
South East Palliative Care Services
South East Palliative Care, University Hospital Waterford, Ireland
South Tees Community Specialist Palliative Care MRC
South Tipperary Hospice Homecare Team
South Tyneside District Hospital Specialist Palliative Care Team, South Tyneside and Sunderland NHS Foundation Trust
Specialist Palliative Care Hospital Team. East Kent University Foundation Trust
Specialist Palliative Care Service South Eastern Health and Social Care Trust
Specialist Palliative Care Team Imperial College Healthcare NHS Trust
Specialist Palliative Care Team, Coventry and Warwickshire Partnership NHS Trust
Specialist Palliative Care Team, West Middlesex University Hospital, Chelsea and Westminster NHSFT
Specialist Palliative Care Service, County Durham and Darlington NHS Foundation Trust

St Ann's Hospice
St Barnabas Hospice, Lincolnshire
St Barnabas House, Worthing
St Bartholomew’s Hospital, Barts Health
St Catherine's Hospice, Preston
St Christopher's
St Clare Hospice, Essex
St Columba's Hospice, Edinburgh
St Gemma's Hospice - Leeds, West Yorkshire.
St Helena
St John of God Murdoch Hospital
St John's Hospice, Doncaster, RDASH
St Johns Hospice, Lancaster
St Leonard's Hospice, York
St Luke's Cheshire Hospice
St Luke's Combined Hospices, Cape Town, South Africa
St Luke's Hospice, Sheffield.
St Luke’s Hospice, Kenton, Harrow covering London boroughs of Brent and Harrow. Ursula Reeve, Director of Patient Services. Cathy Hanrott, Hospice Service Navigator
St Margaret’s Hospice, Somerset.
St Martha's Hospital Palliative Care Team
St Mary's Hospice, Ulverston, Cumbria
St Michael's Hospice, Hastings and Rother
St Oswald's Hospice, Newcastle
St Peter's Hospice
St Richards Hospice
St Wilfrid's Hospice, Chichester
St Wilfrid's Hospice, Eastbourne.
St. Luke's Hospice Community Services South West Essex
Stockport Specialist Palliative Care Service
Strathcarron Hospice
Sue Ryder Leckhampton Court Hospice

Sue Ryder Manorlands Hospice
Susanna Sandöy, Capio Asih Nacka,
Swansea Bay University Health Board Specialist Palliative Care Service (which includes Ty Olwen Hospice)
Symptom Control and Palliative Care Team, Royal Marsden NHS Foundation Trust
Tatiana Chavouzi
Tayside Palliative Care Service
The Arthur Rank Hospice, Cambridge
The Camden, Islington ELiPSe Palliative Care Team
The Hillingdon Hospital Palliative Care team
The Prince of Wales Hospice, Pontefract
The Wisdom Hospice and Medway Community Healthcare
TOPAT Zuyderland MC
Trinity Hospice & Brian House Children's Hospice
Trinity Hospice, Blackpool and Blackpool Teaching Hospitals
Tynedale Hospice at Home
Unità di Cure Palliative - Hospice Casale Monferrato ASL AL Piemonte
University Hospital Lewisham Macmillan Specialist Palliative Care Team, Lewisham and Greenwich NHS Trust
University Teaching Hospital Lusaka Adult Medicine Palliative Care Unit
Waterford Hospice Homecare Team
Weldmar Hospicecare
West Suffolk Hospital NHS Foundation Trust
Western Australia Paediatric & Adolescent Palliative Oncology, Perth Children's Hospital
Weston Hospicecare
Whittington Health Palliative Care Service
Wigan & Leigh Hospice
Wrighinton, Wigan and Leigh NHS Teaching Trust (Supportive and Palliative Care Team)
York Teaching Hospital Foundation Trust
York Teaching Hospitals
Zuercher Lighthouse
Zuzana Kremenova
