# Supplementary material for: The Challenges of Caring for People Dying From COVID-19: A Multinational, Observational Study (CovPall)
Source: J Pain Symptom Manage. 2021 Sep;62(3):460–70. doi: 10.1016/j.jpainsymman.2021.01.138 (PMC7863772; doi:10.1016/j.jpainsymman.2021.01.138)
Supplement: Supplementary file 4 [file mmc4.doc]

**STROBE Statement—Checklist of items that should be included in reports of *cross-sectional studies***

|  | Item No | Recommendation |  |  |  |
| --- | --- | --- | --- | --- | --- |
| **Title and abstract** | 1 | (*a*) Indicate the study’s design with a commonly used term in the title or the abstract |  | Yes |  |
|  |  | (*b*) Provide in the abstract an informative and balanced summary of what was done and what was found |  | Yes |  |
| Introduction | | |  |  |  |
| Background/rationale | 2 | Explain the scientific background and rationale for the investigation being reported |  | Yes |  |
| Objectives | 3 | State specific objectives, including any prespecified hypotheses |  | Yes |  |
| Methods | | |  |  |  |
| Study design | 4 | Present key elements of study design early in the paper |  | Yes |  |
| Setting | 5 | Describe the setting, locations, and relevant dates, including periods of recruitment, exposure, follow-up, and data collection |  | Survey opened on the 23^rd^ of April 2020 and closed on the 31^st^ of July 2020 |  |
| Participants | 6 | (*a*) Give the eligibility criteria, and the sources and methods of selection of participants |  | Yes |  |
| Variables | 7 | Clearly define all outcomes, exposures, predictors, potential confounders, and effect modifiers. Give diagnostic criteria, if applicable |  | Yes |  |
| Data sources/ measurement | 8* | For each variable of interest, give sources of data and details of methods of assessment (measurement). Describe comparability of assessment methods if there is more than one group |  | Yes, textual and quantitative variables described |  |
| Bias | 9 | Describe any efforts to address potential sources of bias |  | Yes –  Page 5  Data were anonymised before analysis |  |
| Study size | 10 | Explain how the study size was arrived at |  | Yes – page 5 |  |
| Quantitative variables | 11 | Explain how quantitative variables were handled in the analyses. If applicable, describe which groupings were chosen and why |  | We used contingency tables, χ2 tests, and correlations in SPSS (v26) and then multivariable logistic regression to explore relationships between variables was done in STATA (v16) |  |
| Statistical methods | 12 | (*a*) Describe all statistical methods, including those used to control for confounding |  | We used contingency tables, χ2 tests, and correlations in SPSS (v26) and then multivariable logistic regression to explore relationships between variables was done in STATA (v16) |  |
|  |  | (*b*) Describe any methods used to examine subgroups and interactions |  | Not applicable |  |
|  |  | (*c*) Explain how missing data were addressed |  | Missing data were not imputed due to limitations inherent in the commonly used approaches for handling missing data |  |
|  |  | (*d*) If applicable, describe analytical methods taking account of sampling strategy |  | Not applicable |  |
|  |  | (*e*) Describe any sensitivity analyses |  | Not applicable |  |
| Results | | |  |  |  |
| Participants | 13* | (a) Report numbers of individuals at each stage of study—eg numbers potentially eligible, examined for eligibility, confirmed eligible, included in the study, completing follow-up, and analysed |  | The numbers potentially eligible could not be determined because this was an online survey. However, a total of 489 questionnaire were commenced, and 477 completed (completion rate 97.5%); of these 15 were duplicates and 2 triplicates of entries with the same name/email; 2 were invalid being from one researcher without a palliative care service, leaving 458 valid responses |  |
|  |  | (b) Give reasons for non-participation at each stage |  | N/A |  |
|  |  | (c) Consider use of a flow diagram |  | N/A |  |
| Descriptive data | 14* | (a) Give characteristics of study participants (eg demographic, clinical, social) and information on exposures and potential confounders |  | Yes – see table 1 and supplementary file I, table S1 for full details |  |
|  |  | (b) Indicate number of participants with missing data for each variable of interest |  | Yes – see table 1 and supplementary file I, table S1 for full details |  |
| Outcome data | 15* | Report numbers of outcome events or summary measures |  | Yes – see table 2 and supplementary file I, tables S4 to S7 |  |
| Main results | 16 | (*a*) Give unadjusted estimates and, if applicable, confounder-adjusted estimates and their precision (eg, 95% confidence interval). Make clear which confounders were adjusted for and why they were included |  | See Table 2 for the multivariate regression analysis. We preselected four dependent variables, presence or not of shortages of: personal protective equipment (PPE), staff, medicines, or other equipment (such as syringe drivers). Independent variables were: country/region, charitable or public management, settings (comprising four settings), experiences with COVID-19 and level of busyness. For each of the four multiple regression analyses we included independent variables showing p<0.10 in univariable analysis, excluding those exhibiting collinearity with independent variables already included if there was variance inflation factor >10 or chi-square test, p < 0.05. |  |
|  |  | (*b*) Report category boundaries when continuous variables were categorized |  | N/A |  |
|  |  | (*c*) If relevant, consider translating estimates of relative risk into absolute risk for a meaningful time period |  | N/A |  |
| Other analyses | 17 | Report other analyses done—eg analyses of subgroups and interactions, and sensitivity analyses |  | Yes  Page 6 (Free text responses) |  |
| Discussion | | |  |  |  |
| Key results | 18 | Summarise key results with reference to study objectives |  | Yes  Pages 10 – 13 |  |
| Limitations | 19 | Discuss limitations of the study, taking into account sources of potential bias or imprecision. Discuss both direction and magnitude of any potential bias |  | Yes |  |
| Interpretation | 20 | Give a cautious overall interpretation of results considering objectives, limitations, multiplicity of analyses, results from similar studies, and other relevant evidence |  | Yes |  |
| Generalisability | 21 | Discuss the generalisability (external validity) of the study results |  | Yes |  |
| Other information | | |  |  |  |
| Funding | 22 | Give the source of funding and the role of the funders for the present study and, if applicable, for the original study on which the present article is based |  | Yes – pages 13 - 14 |  |

*Give information separately for exposed and unexposed groups.

**Note:** An Explanation and Elaboration article discusses each checklist item and gives methodological background and published examples of transparent reporting. The STROBE checklist is best used in conjunction with this article (freely available on the Web sites of PLoS Medicine at http://www.plosmedicine.org/, Annals of Internal Medicine at http://www.annals.org/, and Epidemiology at http://www.epidem.com/). Information on the STROBE Initiative is available at www.strobe-statement.org.
